# Measurement of differential cross sections and charge ratios for *t*-channel single top quark production in proton–proton collisions at $$\sqrt{s}=13$$$$\,\text {Te}\text {V}$$

**DOI:** 10.1140/epjc/s10052-020-7858-1

**Published:** 2020-05-06

**Authors:** A. M. Sirunyan, A. Tumasyan, W. Adam, F. Ambrogi, T. Bergauer, J. Brandstetter, M. Dragicevic, J. Erö, A. Escalante Del Valle, M. Flechl, R. Frühwirth, M. Jeitler, N. Krammer, I. Krätschmer, D. Liko, T. Madlener, I. Mikulec, N. Rad, J. Schieck, R. Schöfbeck, M. Spanring, D. Spitzbart, W. Waltenberger, C.-E. Wulz, M. Zarucki, V. Drugakov, V. Mossolov, J. Suarez Gonzalez, M. R. Darwish, E. A. De Wolf, D. Di Croce, X. Janssen, J. Lauwers, A. Lelek, M. Pieters, H. Rejeb Sfar, H. Van Haevermaet, P. Van Mechelen, S. Van Putte, N. Van Remortel, F. Blekman, E. S. Bols, S. S. Chhibra, J. D’Hondt, J. De Clercq, D. Lontkovskyi, S. Lowette, I. Marchesini, S. Moortgat, L. Moreels, Q. Python, K. Skovpen, S. Tavernier, W. Van Doninck, P. Van Mulders, I. Van Parijs, D. Beghin, B. Bilin, H. Brun, B. Clerbaux, G. De Lentdecker, H. Delannoy, B. Dorney, L. Favart, A. Grebenyuk, A. K. Kalsi, J. Luetic, A. Popov, N. Postiau, E. Starling, L. Thomas, C. Vander Velde, P. Vanlaer, D. Vannerom, Q. Wang, T. Cornelis, D. Dobur, I. Khvastunov, C. Roskas, D. Trocino, M. Tytgat, W. Verbeke, B. Vermassen, M. Vit, N. Zaganidis, O. Bondu, G. Bruno, C. Caputo, P. David, C. Delaere, M. Delcourt, A. Giammanco, V. Lemaitre, A. Magitteri, J. Prisciandaro, A. Saggio, M. Vidal Marono, P. Vischia, J. Zobec, F. L. Alves, G. A. Alves, G. Correia Silva, C. Hensel, A. Moraes, P. Rebello Teles, E. Belchior Batista Das Chagas, W. Carvalho, J. Chinellato, E. Coelho, E. M. Da Costa, G. G. Da Silveira, D. De Jesus Damiao, C. De Oliveira Martins, S. Fonseca De Souza, L. M. Huertas Guativa, H. Malbouisson, J. Martins, D. Matos Figueiredo, M. Medina Jaime, M. Melo De Almeida, C. Mora Herrera, L. Mundim, H. Nogima, W. L. Prado Da Silva, L. J. Sanchez Rosas, A. Santoro, A. Sznajder, M. Thiel, E. J. Tonelli Manganote, F. Torres Da Silva De Araujo, A. Vilela Pereira, S. Ahuja, C. A. Bernardes, L. Calligaris, T. R. Fernandez Perez Tomei, E. M. Gregores, D. S. Lemos, P. G. Mercadante, S. F. Novaes, SandraS. Padula, A. Aleksandrov, G. Antchev, R. Hadjiiska, P. Iaydjiev, A. Marinov, M. Misheva, M. Rodozov, M. Shopova, G. Sultanov, M. Bonchev, A. Dimitrov, T. Ivanov, L. Litov, B. Pavlov, P. Petkov, W. Fang, X. Gao, L. Yuan, Z. Hu, Y. Wang, M. Ahmad, G. M. Chen, H. S. Chen, M. Chen, C. H. Jiang, D. Leggat, H. Liao, Z. Liu, S. M. Shaheen, A. Spiezia, J. Tao, E. Yazgan, H. Zhang, S. Zhang, J. Zhao, A. Agapitos, Y. Ban, G. Chen, A. Levin, J. Li, L. Li, Q. Li, Y. Mao, S. J. Qian, D. Wang, C. Avila, A. Cabrera, L. F. Chaparro Sierra, C. Florez, C. F. González Hernández, M. A. Segura Delgado, J. Mejia Guisao, J. D. Ruiz Alvarez, C. A. Salazar González, N. Vanegas Arbelaez, D. Giljanović, N. Godinovic, D. Lelas, I. Puljak, T. Sculac, Z. Antunovic, M. Kovac, V. Brigljevic, S. Ceci, D. Ferencek, K. Kadija, B. Mesic, M. Roguljic, A. Starodumov, T. Susa, M. W. Ather, A. Attikis, E. Erodotou, A. Ioannou, M. Kolosova, S. Konstantinou, G. Mavromanolakis, J. Mousa, C. Nicolaou, F. Ptochos, P. A. Razis, H. Rykaczewski, D. Tsiakkouri, M. Finger, M. Finger, A. Kveton, J. Tomsa, E. Ayala, E. Carrera Jarrin, H. Abdalla, A. A. Abdelalim, S. Bhowmik, A. Carvalho Antunes De Oliveira, R. K. Dewanjee, K. Ehataht, M. Kadastik, M. Raidal, C. Veelken, P. Eerola, L. Forthomme, H. Kirschenmann, K. Osterberg, M. Voutilainen, F. Garcia, J. Havukainen, J. K. Heikkilä, T. Järvinen, V. Karimäki, R. Kinnunen, T. Lampén, K. Lassila-Perini, S. Laurila, S. Lehti, T. Lindén, P. Luukka, T. Mäenpää, H. Siikonen, E. Tuominen, J. Tuominiemi, T. Tuuva, M. Besancon, F. Couderc, M. Dejardin, D. Denegri, B. Fabbro, J. L. Faure, F. Ferri, S. Ganjour, A. Givernaud, P. Gras, G. Hamel de Monchenault, P. Jarry, C. Leloup, E. Locci, J. Malcles, J. Rander, A. Rosowsky, M. Ö. Sahin, A. Savoy-Navarro, M. Titov, C. Amendola, F. Beaudette, P. Busson, C. Charlot, B. Diab, G. Falmagne, R. Granier de Cassagnac, I. Kucher, A. Lobanov, C. Martin Perez, M. Nguyen, C. Ochando, P. Paganini, J. Rembser, R. Salerno, J. B. Sauvan, Y. Sirois, A. Zabi, A. Zghiche, J.-L. Agram, J. Andrea, D. Bloch, G. Bourgatte, J.-M. Brom, E. C. Chabert, C. Collard, E. Conte, J.-C. Fontaine, D. Gelé, U. Goerlach, M. Jansová, A.-C. Le Bihan, N. Tonon, P. Van Hove, S. Gadrat, S. Beauceron, C. Bernet, G. Boudoul, C. Camen, N. Chanon, R. Chierici, D. Contardo, P. Depasse, H. El Mamouni, J. Fay, S. Gascon, M. Gouzevitch, B. Ille, Sa. Jain, F. Lagarde, I. B. Laktineh, H. Lattaud, M. Lethuillier, L. Mirabito, S. Perries, V. Sordini, G. Touquet, M. Vander Donckt, S. Viret, T. Toriashvili, Z. Tsamalaidze, C. Autermann, L. Feld, M. K. Kiesel, K. Klein, M. Lipinski, D. Meuser, A. Pauls, M. Preuten, M. P. Rauch, C. Schomakers, J. Schulz, M. Teroerde, B. Wittmer, A. Albert, M. Erdmann, S. Erdweg, T. Esch, B. Fischer, R. Fischer, S. Ghosh, T. Hebbeker, K. Hoepfner, H. Keller, L. Mastrolorenzo, M. Merschmeyer, A. Meyer, P. Millet, G. Mocellin, S. Mondal, S. Mukherjee, D. Noll, A. Novak, T. Pook, A. Pozdnyakov, T. Quast, M. Radziej, Y. Rath, H. Reithler, M. Rieger, J. Roemer, A. Schmidt, S. C. Schuler, A. Sharma, S. Thüer, S. Wiedenbeck, G. Flügge, W. Haj Ahmad, O. Hlushchenko, T. Kress, T. Müller, A. Nehrkorn, A. Nowack, C. Pistone, O. Pooth, D. Roy, H. Sert, A. Stahl, M. Aldaya Martin, P. Asmuss, I. Babounikau, H. Bakhshiansohi, K. Beernaert, O. Behnke, U. Behrens, A. Bermúdez Martínez, D. Bertsche, A. A. Bin Anuar, K. Borras, V. Botta, A. Campbell, A. Cardini, P. Connor, S. Consuegra Rodríguez, C. Contreras-Campana, V. Danilov, A. De Wit, M. M. Defranchis, C. Diez Pardos, D. Domínguez Damiani, G. Eckerlin, D. Eckstein, T. Eichhorn, A. Elwood, E. Eren, E. Gallo, A. Geiser, J. M. Grados Luyando, A. Grohsjean, M. Guthoff, M. Haranko, A. Harb, A. Jafari, N. Z. Jomhari, H. Jung, A. Kasem, M. Kasemann, H. Kaveh, J. Keaveney, C. Kleinwort, J. Knolle, D. Krücker, W. Lange, T. Lenz, J. Leonard, J. Lidrych, K. Lipka, W. Lohmann, R. Mankel, I.-A. Melzer-Pellmann, A. B. Meyer, M. Meyer, M. Missiroli, G. Mittag, J. Mnich, A. Mussgiller, V. Myronenko, D. Pérez Adán, S. K. Pflitsch, D. Pitzl, A. Raspereza, A. Saibel, M. Savitskyi, V. Scheurer, P. Schütze, C. Schwanenberger, R. Shevchenko, A. Singh, H. Tholen, O. Turkot, A. Vagnerini, M. Van De Klundert, G. P. Van Onsem, R. Walsh, Y. Wen, K. Wichmann, C. Wissing, O. Zenaiev, R. Zlebcik, R. Aggleton, S. Bein, L. Benato, A. Benecke, V. Blobel, T. Dreyer, A. Ebrahimi, A. Fröhlich, C. Garbers, E. Garutti, D. Gonzalez, P. Gunnellini, J. Haller, A. Hinzmann, A. Karavdina, G. Kasieczka, R. Klanner, R. Kogler, N. Kovalchuk, S. Kurz, V. Kutzner, J. Lange, T. Lange, A. Malara, D. Marconi, J. Multhaup, M. Niedziela, C. E. N. Niemeyer, D. Nowatschin, A. Perieanu, A. Reimers, O. Rieger, C. Scharf, P. Schleper, S. Schumann, J. Schwandt, J. Sonneveld, H. Stadie, G. Steinbrück, F. M. Stober, M. Stöver, B. Vormwald, I. Zoi, M. Akbiyik, C. Barth, M. Baselga, S. Baur, T. Berger, E. Butz, R. Caspart, T. Chwalek, W. De Boer, A. Dierlamm, K. El Morabit, N. Faltermann, M. Giffels, P. Goldenzweig, A. Gottmann, M. A. Harrendorf, F. Hartmann, U. Husemann, S. Kudella, S. Mitra, M. U. Mozer, Th. Müller, M. Musich, A. Nürnberg, G. Quast, K. Rabbertz, M. Schröder, I. Shvetsov, H. J. Simonis, R. Ulrich, M. Weber, C. Wöhrmann, R. Wolf, G. Anagnostou, P. Asenov, G. Daskalakis, T. Geralis, A. Kyriakis, D. Loukas, G. Paspalaki, M. Diamantopoulou, G. Karathanasis, P. Kontaxakis, A. Panagiotou, I. Papavergou, N. Saoulidou, A. Stakia, K. Theofilatos, K. Vellidis, G. Bakas, K. Kousouris, I. Papakrivopoulos, G. Tsipolitis, I. Evangelou, C. Foudas, P. Gianneios, P. Katsoulis, P. Kokkas, S. Mallios, K. Manitara, N. Manthos, I. Papadopoulos, J. Strologas, F. A. Triantis, D. Tsitsonis, M. Bartók, M. Csanad, P. Major, K. Mandal, A. Mehta, M. I. Nagy, G. Pasztor, O. Surányi, G. I. Veres, G. Bencze, C. Hajdu, D. Horvath, F. Sikler, T. Á. Vámi, V. Veszpremi, G. Vesztergombi, N. Beni, S. Czellar, J. Karancsi, A. Makovec, J. Molnar, Z. Szillasi, P. Raics, D. Teyssier, Z. L. Trocsanyi, B. Ujvari, T. Csorgo, W. J. Metzger, F. Nemes, T. Novak, S. Choudhury, J. R. Komaragiri, P. C. Tiwari, S. Bahinipati, C. Kar, G. Kole, P. Mal, V. K. Muraleedharan Nair Bindhu, A. Nayak, D. K. Sahoo, S. K. Swain, S. Bansal, S. B. Beri, V. Bhatnagar, S. Chauhan, R. Chawla, N. Dhingra, R. Gupta, A. Kaur, M. Kaur, S. Kaur, P. Kumari, M. Lohan, M. Meena, K. Sandeep, S. Sharma, J. B. Singh, A. K. Virdi, G. Walia, A. Bhardwaj, B. C. Choudhary, R. B. Garg, M. Gola, S. Keshri, Ashok Kumar, S. Malhotra, M. Naimuddin, P. Priyanka, K. Ranjan, Aashaq Shah, R. Sharma, R. Bhardwaj, M. Bharti, R. Bhattacharya, S. Bhattacharya, U. Bhawandeep, D. Bhowmik, S. Dey, S. Dutta, S. Ghosh, M. Maity, K. Mondal, S. Nandan, A. Purohit, P. K. Rout, G. Saha, S. Sarkar, T. Sarkar, M. Sharan, B. Singh, S. Thakur, P. K. Behera, P. Kalbhor, A. Muhammad, P. R. Pujahari, A. Sharma, A. K. Sikdar, R. Chudasama, D. Dutta, V. Jha, V. Kumar, D. K. Mishra, P. K. Netrakanti, L. M. Pant, P. Shukla, T. Aziz, M. A. Bhat, S. Dugad, G. B. Mohanty, N. Sur, RavindraKumar Verma, S. Banerjee, S. Bhattacharya, S. Chatterjee, P. Das, M. Guchait, S. Karmakar, S. Kumar, G. Majumder, K. Mazumdar, N. Sahoo, S. Sawant, S. Chauhan, S. Dube, V. Hegde, A. Kapoor, K. Kothekar, S. Pandey, A. Rane, A. Rastogi, S. Sharma, S. Chenarani, E. Eskandari Tadavani, S. M. Etesami, M. Khakzad, M. Mohammadi Najafabadi, M. Naseri, F. Rezaei Hosseinabadi, M. Felcini, M. Grunewald, M. Abbrescia, C. Calabria, A. Colaleo, D. Creanza, L. Cristella, N. De Filippis, M. De Palma, A. Di Florio, L. Fiore, A. Gelmi, G. Iaselli, M. Ince, S. Lezki, G. Maggi, M. Maggi, G. Miniello, S. My, S. Nuzzo, A. Pompili, G. Pugliese, R. Radogna, A. Ranieri, G. Selvaggi, L. Silvestris, R. Venditti, P. Verwilligen, G. Abbiendi, C. Battilana, D. Bonacorsi, L. Borgonovi, S. Braibant-Giacomelli, R. Campanini, P. Capiluppi, A. Castro, F. R. Cavallo, C. Ciocca, G. Codispoti, M. Cuffiani, G. M. Dallavalle, F. Fabbri, A. Fanfani, E. Fontanesi, P. Giacomelli, C. Grandi, L. Guiducci, F. Iemmi, S. Lo Meo, S. Marcellini, G. Masetti, F. L. Navarria, A. Perrotta, F. Primavera, A. M. Rossi, T. Rovelli, G. P. Siroli, N. Tosi, S. Albergo, S. Costa, A. Di Mattia, R. Potenza, A. Tricomi, C. Tuve, G. Barbagli, R. Ceccarelli, K. Chatterjee, V. Ciulli, C. Civinini, R. D’Alessandro, E. Focardi, G. Latino, P. Lenzi, M. Meschini, S. Paoletti, G. Sguazzoni, D. Strom, L. Viliani, L. Benussi, S. Bianco, D. Piccolo, M. Bozzo, F. Ferro, R. Mulargia, E. Robutti, S. Tosi, A. Benaglia, A. Beschi, F. Brivio, V. Ciriolo, S. Di Guida, M. E. Dinardo, P. Dini, S. Fiorendi, S. Gennai, A. Ghezzi, P. Govoni, L. Guzzi, M. Malberti, S. Malvezzi, D. Menasce, F. Monti, L. Moroni, G. Ortona, M. Paganoni, D. Pedrini, S. Ragazzi, T. Tabarelli de Fatis, D. Zuolo, S. Buontempo, N. Cavallo, A. De Iorio, A. Di Crescenzo, F. Fabozzi, F. Fienga, G. Galati, A. O. M. Iorio, L. Lista, S. Meola, P. Paolucci, B. Rossi, C. Sciacca, E. Voevodina, P. Azzi, N. Bacchetta, A. Boletti, A. Bragagnolo, R. Carlin, P. Checchia, P. De Castro Manzano, T. Dorigo, U. Dosselli, F. Gasparini, U. Gasparini, A. Gozzelino, S. Y. Hoh, P. Lujan, M. Margoni, A. T. Meneguzzo, J. Pazzini, N. Pozzobon, M. Presilla, P. Ronchese, R. Rossin, F. Simonetto, A. Tiko, M. Tosi, M. Zanetti, P. Zotto, G. Zumerle, A. Braghieri, P. Montagna, S. P. Ratti, V. Re, M. Ressegotti, C. Riccardi, P. Salvini, I. Vai, P. Vitulo, M. Biasini, G. M. Bilei, C. Cecchi, D. Ciangottini, L. Fanò, P. Lariccia, R. Leonardi, E. Manoni, G. Mantovani, V. Mariani, M. Menichelli, A. Rossi, A. Santocchia, D. Spiga, K. Androsov, P. Azzurri, G. Bagliesi, V. Bertacchi, L. Bianchini, T. Boccali, R. Castaldi, M. A. Ciocci, R. Dell’Orso, G. Fedi, L. Giannini, A. Giassi, M. T. Grippo, F. Ligabue, E. Manca, G. Mandorli, A. Messineo, F. Palla, A. Rizzi, G. Rolandi, S. Roy Chowdhury, A. Scribano, P. Spagnolo, R. Tenchini, G. Tonelli, N. Turini, A. Venturi, P. G. Verdini, F. Cavallari, M. Cipriani, D. Del Re, E. Di Marco, M. Diemoz, E. Longo, B. Marzocchi, P. Meridiani, G. Organtini, F. Pandolfi, R. Paramatti, C. Quaranta, S. Rahatlou, C. Rovelli, F. Santanastasio, L. Soffi, N. Amapane, R. Arcidiacono, S. Argiro, M. Arneodo, N. Bartosik, R. Bellan, C. Biino, A. Cappati, N. Cartiglia, S. Cometti, M. Costa, R. Covarelli, N. Demaria, B. Kiani, C. Mariotti, S. Maselli, E. Migliore, V. Monaco, E. Monteil, M. Monteno, M. M. Obertino, L. Pacher, N. Pastrone, M. Pelliccioni, G. L. Pinna Angioni, A. Romero, M. Ruspa, R. Sacchi, R. Salvatico, V. Sola, A. Solano, D. Soldi, A. Staiano, S. Belforte, V. Candelise, M. Casarsa, F. Cossutti, A. Da Rold, G. Della Ricca, F. Vazzoler, A. Zanetti, B. Kim, D. H. Kim, G. N. Kim, M. S. Kim, J. Lee, S. W. Lee, C. S. Moon, Y. D. Oh, S. I. Pak, S. Sekmen, D. C. Son, Y. C. Yang, H. Kim, D. H. Moon, G. Oh, B. Francois, T. J. Kim, J. Park, S. Cho, S. Choi, Y. Go, D. Gyun, S. Ha, B. Hong, K. Lee, K. S. Lee, J. Lim, J. Park, S. K. Park, Y. Roh, J. Goh, H. S. Kim, J. Almond, J. H. Bhyun, J. Choi, S. Jeon, J. Kim, J. S. Kim, H. Lee, K. Lee, S. Lee, K. Nam, M. Oh, S. B. Oh, B. C. Radburn-Smith, U. K. Yang, H. D. Yoo, I. Yoon, G. B. Yu, D. Jeon, H. Kim, J. H. Kim, J. S. H. Lee, I. C. Park, I. Watson, Y. Choi, C. Hwang, Y. Jeong, J. Lee, Y. Lee, I. Yu, V. Veckalns, V. Dudenas, A. Juodagalvis, J. Vaitkus, Z. A. Ibrahim, F. Mohamad Idris, W. A. T. Wan Abdullah, M. N. Yusli, Z. Zolkapli, J. F. Benitez, A. Castaneda Hernandez, J. A. Murillo Quijada, L. Valencia Palomo, H. Castilla-Valdez, E. De La Cruz-Burelo, I. Heredia-De La Cruz, R. Lopez-Fernandez, A. Sanchez-Hernandez, S. Carrillo Moreno, C. Oropeza Barrera, M. Ramirez-Garcia, F. Vazquez Valencia, J. Eysermans, I. Pedraza, H. A. Salazar Ibarguen, C. Uribe Estrada, A. Morelos Pineda, N. Raicevic, D. Krofcheck, S. Bheesette, P. H. Butler, A. Ahmad, M. Ahmad, Q. Hassan, H. R. Hoorani, W. A. Khan, M. A. Shah, M. Shoaib, M. Waqas, V. Avati, L. Grzanka, M. Malawski, H. Bialkowska, M. Bluj, B. Boimska, M. Górski, M. Kazana, M. Szleper, P. Zalewski, K. Bunkowski, A. Byszuk, K. Doroba, A. Kalinowski, M. Konecki, J. Krolikowski, M. Misiura, M. Olszewski, A. Pyskir, M. Walczak, M. Araujo, P. Bargassa, D. Bastos, A. Di Francesco, P. Faccioli, B. Galinhas, M. Gallinaro, J. Hollar, N. Leonardo, J. Seixas, K. Shchelina, G. Strong, O. Toldaiev, J. Varela, S. Afanasiev, P. Bunin, M. Gavrilenko, I. Golutvin, I. Gorbunov, A. Kamenev, V. Karjavine, A. Lanev, A. Malakhov, V. Matveev, P. Moisenz, V. Palichik, V. Perelygin, M. Savina, S. Shmatov, S. Shulha, N. Skatchkov, V. Smirnov, N. Voytishin, A. Zarubin, L. Chtchipounov, V. Golovtsov, Y. Ivanov, V. Kim, E. Kuznetsova, P. Levchenko, V. Murzin, V. Oreshkin, I. Smirnov, D. Sosnov, V. Sulimov, L. Uvarov, A. Vorobyev, Yu. Andreev, A. Dermenev, S. Gninenko, N. Golubev, A. Karneyeu, M. Kirsanov, N. Krasnikov, A. Pashenkov, D. Tlisov, A. Toropin, V. Epshteyn, V. Gavrilov, N. Lychkovskaya, A. Nikitenko, V. Popov, I. Pozdnyakov, G. Safronov, A. Spiridonov, A. Stepennov, M. Toms, E. Vlasov, A. Zhokin, T. Aushev, M. Chadeeva, P. Parygin, D. Philippov, E. Popova, V. Rusinov, V. Andreev, M. Azarkin, I. Dremin, M. Kirakosyan, A. Terkulov, A. Baskakov, A. Belyaev, E. Boos, V. Bunichev, M. Dubinin, L. Dudko, V. Klyukhin, N. Korneeva, I. Lokhtin, S. Obraztsov, M. Perfilov, V. Savrin, P. Volkov, A. Barnyakov, V. Blinov, T. Dimova, L. Kardapoltsev, Y. Skovpen, I. Azhgirey, I. Bayshev, S. Bitioukov, V. Kachanov, D. Konstantinov, P. Mandrik, V. Petrov, R. Ryutin, S. Slabospitskii, A. Sobol, S. Troshin, N. Tyurin, A. Uzunian, A. Volkov, A. Babaev, A. Iuzhakov, V. Okhotnikov, V. Borchsh, V. Ivanchenko, E. Tcherniaev, P. Adzic, P. Cirkovic, D. Devetak, M. Dordevic, P. Milenovic, J. Milosevic, M. Stojanovic, M. Aguilar-Benitez, J. Alcaraz Maestre, A. Álvarez Fernández, I. Bachiller, M. Barrio Luna, J. A. Brochero Cifuentes, C. A. Carrillo Montoya, M. Cepeda, M. Cerrada, N. Colino, B. De La Cruz, A. Delgado Peris, C. Fernandez Bedoya, J. P. Fernández Ramos, J. Flix, M. C. Fouz, O. Gonzalez Lopez, S. Goy Lopez, J. M. Hernandez, M. I. Josa, D. Moran, Á. Navarro Tobar, A. Pérez-Calero Yzquierdo, J. Puerta Pelayo, I. Redondo, L. Romero, S. Sánchez Navas, M. S. Soares, A. Triossi, C. Willmott, C. Albajar, J. F. de Trocóniz, B. Alvarez Gonzalez, J. Cuevas, C. Erice, J. Fernandez Menendez, S. Folgueras, I. Gonzalez Caballero, J. R. González Fernández, E. Palencia Cortezon, V. Rodríguez Bouza, S. Sanchez Cruz, I. J. Cabrillo, A. Calderon, B. Chazin Quero, J. Duarte Campderros, M. Fernandez, P. J. Fernández Manteca, A. García Alonso, G. Gomez, C. Martinez Rivero, P. Martinez Ruiz del Arbol, F. Matorras, J. Piedra Gomez, C. Prieels, T. Rodrigo, A. Ruiz-Jimeno, L. Russo, L. Scodellaro, N. Trevisani, I. Vila, J. M. Vizan Garcia, K. Malagalage, W. G. D. Dharmaratna, N. Wickramage, D. Abbaneo, B. Akgun, E. Auffray, G. Auzinger, J. Baechler, P. Baillon, A. H. Ball, D. Barney, J. Bendavid, M. Bianco, A. Bocci, E. Bossini, C. Botta, E. Brondolin, T. Camporesi, A. Caratelli, G. Cerminara, E. Chapon, G. Cucciati, D. d’Enterria, A. Dabrowski, N. Daci, V. Daponte, A. David, O. Davignon, A. De Roeck, N. Deelen, M. Deile, M. Dobson, M. Dünser, N. Dupont, A. Elliott-Peisert, F. Fallavollita, D. Fasanella, G. Franzoni, J. Fulcher, W. Funk, S. Giani, D. Gigi, A. Gilbert, K. Gill, F. Glege, M. Gruchala, M. Guilbaud, D. Gulhan, J. Hegeman, C. Heidegger, Y. Iiyama, V. Innocente, P. Janot, O. Karacheban, J. Kaspar, J. Kieseler, M. Krammer, C. Lange, P. Lecoq, C. Lourenço, L. Malgeri, M. Mannelli, A. Massironi, F. Meijers, J. A. Merlin, S. Mersi, E. Meschi, F. Moortgat, M. Mulders, J. Ngadiuba, S. Nourbakhsh, S. Orfanelli, L. Orsini, F. Pantaleo, L. Pape, E. Perez, M. Peruzzi, A. Petrilli, G. Petrucciani, A. Pfeiffer, M. Pierini, F. M. Pitters, D. Rabady, A. Racz, M. Rovere, H. Sakulin, C. Schäfer, C. Schwick, M. Selvaggi, A. Sharma, P. Silva, W. Snoeys, P. Sphicas, J. Steggemann, V. R. Tavolaro, D. Treille, A. Tsirou, A. Vartak, M. Verzetti, W. D. Zeuner, L. Caminada, K. Deiters, W. Erdmann, R. Horisberger, Q. Ingram, H. C. Kaestli, D. Kotlinski, U. Langenegger, T. Rohe, S. A. Wiederkehr, M. Backhaus, P. Berger, N. Chernyavskaya, G. Dissertori, M. Dittmar, M. Donegà, C. Dorfer, T. A. Gómez Espinosa, C. Grab, D. Hits, T. Klijnsma, W. Lustermann, R. A. Manzoni, M. Marionneau, M. T. Meinhard, F. Micheli, P. Musella, F. Nessi-Tedaldi, F. Pauss, G. Perrin, L. Perrozzi, S. Pigazzini, M. Reichmann, C. Reissel, T. Reitenspiess, D. Ruini, D. A. Sanz Becerra, M. Schönenberger, L. Shchutska, M. L. Vesterbacka Olsson, R. Wallny, D. H. Zhu, T. K. Aarrestad, C. Amsler, D. Brzhechko, M. F. Canelli, A. De Cosa, R. Del Burgo, S. Donato, B. Kilminster, S. Leontsinis, V. M. Mikuni, I. Neutelings, G. Rauco, P. Robmann, D. Salerno, K. Schweiger, C. Seitz, Y. Takahashi, S. Wertz, A. Zucchetta, T. H. Doan, C. M. Kuo, W. Lin, A. Roy, S. S. Yu, P. Chang, Y. Chao, K. F. Chen, P. H. Chen, W.-S. Hou, Y. y. Li, R.-S. Lu, E. Paganis, A. Psallidas, A. Steen, B. Asavapibhop, C. Asawatangtrakuldee, N. Srimanobhas, N. Suwonjandee, A. Bat, F. Boran, S. Cerci, S. Damarseckin, Z. S. Demiroglu, F. Dolek, C. Dozen, I. Dumanoglu, E. Eskut, G. Gokbulut, EmineGurpinar Guler, Y. Guler, I. Hos, C. Isik, E. E. Kangal, O. Kara, A. Kayis Topaksu, U. Kiminsu, M. Oglakci, G. Onengut, K. Ozdemir, A. E. Simsek, B. Tali, U. G. Tok, S. Turkcapar, I. S. Zorbakir, C. Zorbilmez, B. Isildak, G. Karapinar, M. Yalvac, I. O. Atakisi, E. Gülmez, M. Kaya, O. Kaya, B. Kaynak, Ö. Özçelik, S. Tekten, E. A. Yetkin, A. Cakir, K. Cankocak, Y. Komurcu, S. Sen, S. Ozkorucuklu, B. Grynyov, L. Levchuk, F. Ball, E. Bhal, S. Bologna, J. J. Brooke, D. Burns, E. Clement, D. Cussans, H. Flacher, J. Goldstein, G. P. Heath, H. F. Heath, L. Kreczko, S. Paramesvaran, B. Penning, T. Sakuma, S. Seif El Nasr-Storey, D. Smith, V. J. Smith, J. Taylor, A. Titterton, K. W. Bell, A. Belyaev, C. Brew, R. M. Brown, D. Cieri, D. J. A. Cockerill, J. A. Coughlan, K. Harder, S. Harper, J. Linacre, K. Manolopoulos, D. M. Newbold, E. Olaiya, D. Petyt, T. Reis, T. Schuh, C. H. Shepherd-Themistocleous, A. Thea, I. R. Tomalin, T. Williams, W. J. Womersley, R. Bainbridge, P. Bloch, J. Borg, S. Breeze, O. Buchmuller, A. Bundock, GurpreetSingh CHAHAL, D. Colling, P. Dauncey, G. Davies, M. Della Negra, R. Di Maria, P. Everaerts, G. Hall, G. Iles, T. James, M. Komm, C. Laner, L. Lyons, A.-M. Magnan, S. Malik, A. Martelli, V. Milosevic, J. Nash, V. Palladino, M. Pesaresi, D. M. Raymond, A. Richards, A. Rose, E. Scott, C. Seez, A. Shtipliyski, M. Stoye, T. Strebler, S. Summers, A. Tapper, K. Uchida, T. Virdee, N. Wardle, D. Winterbottom, J. Wright, A. G. Zecchinelli, S. C. Zenz, J. E. Cole, P. R. Hobson, A. Khan, P. Kyberd, C. K. Mackay, A. Morton, I. D. Reid, L. Teodorescu, S. Zahid, K. Call, J. Dittmann, K. Hatakeyama, C. Madrid, B. McMaster, N. Pastika, C. Smith, R. Bartek, A. Dominguez, R. Uniyal, A. Buccilli, S. I. Cooper, C. Henderson, P. Rumerio, C. West, D. Arcaro, T. Bose, Z. Demiragli, D. Gastler, S. Girgis, D. Pinna, C. Richardson, J. Rohlf, D. Sperka, I. Suarez, L. Sulak, D. Zou, G. Benelli, B. Burkle, X. Coubez, D. Cutts, Y. t. Duh, M. Hadley, J. Hakala, U. Heintz, J. M. Hogan, K. H. M. Kwok, E. Laird, G. Landsberg, J. Lee, Z. Mao, M. Narain, S. Sagir, R. Syarif, E. Usai, D. Yu, R. Band, C. Brainerd, R. Breedon, M. Calderon De La Barca Sanchez, M. Chertok, J. Conway, R. Conway, P. T. Cox, R. Erbacher, C. Flores, G. Funk, F. Jensen, W. Ko, O. Kukral, R. Lander, M. Mulhearn, D. Pellett, J. Pilot, M. Shi, D. Stolp, D. Taylor, K. Tos, M. Tripathi, Z. Wang, F. Zhang, M. Bachtis, C. Bravo, R. Cousins, A. Dasgupta, A. Florent, J. Hauser, M. Ignatenko, N. Mccoll, W. A. Nash, S. Regnard, D. Saltzberg, C. Schnaible, B. Stone, V. Valuev, K. Burt, R. Clare, J. W. Gary, S. M. A. Ghiasi Shirazi, G. Hanson, G. Karapostoli, E. Kennedy, O. R. Long, M. Olmedo Negrete, M. I. Paneva, W. Si, L. Wang, H. Wei, S. Wimpenny, B. R. Yates, Y. Zhang, J. G. Branson, P. Chang, S. Cittolin, M. Derdzinski, R. Gerosa, D. Gilbert, B. Hashemi, D. Klein, V. Krutelyov, J. Letts, M. Masciovecchio, S. May, S. Padhi, M. Pieri, V. Sharma, M. Tadel, F. Würthwein, A. Yagil, G. Zevi Della Porta, N. Amin, R. Bhandari, C. Campagnari, M. Citron, V. Dutta, M. Franco Sevilla, L. Gouskos, J. Incandela, B. Marsh, H. Mei, A. Ovcharova, H. Qu, J. Richman, U. Sarica, D. Stuart, S. Wang, J. Yoo, D. Anderson, A. Bornheim, O. Cerri, I. Dutta, J. M. Lawhorn, N. Lu, J. Mao, H. B. Newman, T. Q. Nguyen, J. Pata, M. Spiropulu, J. R. Vlimant, S. Xie, Z. Zhang, R. Y. Zhu, M. B. Andrews, T. Ferguson, T. Mudholkar, M. Paulini, M. Sun, I. Vorobiev, M. Weinberg, J. P. Cumalat, W. T. Ford, A. Johnson, E. MacDonald, T. Mulholland, R. Patel, A. Perloff, K. Stenson, K. A. Ulmer, S. R. Wagner, J. Alexander, J. Chaves, Y. Cheng, J. Chu, A. Datta, A. Frankenthal, K. Mcdermott, N. Mirman, J. R. Patterson, D. Quach, A. Rinkevicius, A. Ryd, S. M. Tan, Z. Tao, J. Thom, P. Wittich, M. Zientek, S. Abdullin, M. Albrow, M. Alyari, G. Apollinari, A. Apresyan, A. Apyan, S. Banerjee, L. A. T. Bauerdick, A. Beretvas, J. Berryhill, P. C. Bhat, K. Burkett, J. N. Butler, A. Canepa, G. B. Cerati, H. W. K. Cheung, F. Chlebana, M. Cremonesi, J. Duarte, V. D. Elvira, J. Freeman, Z. Gecse, E. Gottschalk, L. Gray, D. Green, S. Grünendahl, O. Gutsche, AllisonReinsvold Hall, J. Hanlon, R. M. Harris, S. Hasegawa, R. Heller, J. Hirschauer, B. Jayatilaka, S. Jindariani, M. Johnson, U. Joshi, B. Klima, M. J. Kortelainen, B. Kreis, S. Lammel, J. Lewis, D. Lincoln, R. Lipton, M. Liu, T. Liu, J. Lykken, K. Maeshima, J. M. Marraffino, D. Mason, P. McBride, P. Merkel, S. Mrenna, S. Nahn, V. O’Dell, V. Papadimitriou, K. Pedro, C. Pena, G. Rakness, F. Ravera, L. Ristori, B. Schneider, E. Sexton-Kennedy, N. Smith, A. Soha, W. J. Spalding, L. Spiegel, S. Stoynev, J. Strait, N. Strobbe, L. Taylor, S. Tkaczyk, N. V. Tran, L. Uplegger, E. W. Vaandering, C. Vernieri, M. Verzocchi, R. Vidal, M. Wang, H. A. Weber, D. Acosta, P. Avery, P. Bortignon, D. Bourilkov, A. Brinkerhoff, L. Cadamuro, A. Carnes, V. Cherepanov, D. Curry, F. Errico, R. D. Field, S. V. Gleyzer, B. M. Joshi, M. Kim, J. Konigsberg, A. Korytov, K. H. Lo, P. Ma, K. Matchev, N. Menendez, G. Mitselmakher, D. Rosenzweig, K. Shi, J. Wang, S. Wang, X. Zuo, Y. R. Joshi, T. Adams, A. Askew, S. Hagopian, V. Hagopian, K. F. Johnson, R. Khurana, T. Kolberg, G. Martinez, T. Perry, H. Prosper, C. Schiber, R. Yohay, J. Zhang, M. M. Baarmand, V. Bhopatkar, M. Hohlmann, D. Noonan, M. Rahmani, M. Saunders, F. Yumiceva, M. R. Adams, L. Apanasevich, D. Berry, R. R. Betts, R. Cavanaugh, X. Chen, S. Dittmer, O. Evdokimov, C. E. Gerber, D. A. Hangal, D. J. Hofman, K. Jung, C. Mills, T. Roy, M. B. Tonjes, N. Varelas, H. Wang, X. Wang, Z. Wu, M. Alhusseini, B. Bilki, W. Clarida, K. Dilsiz, S. Durgut, R. P. Gandrajula, M. Haytmyradov, V. Khristenko, O. K. Köseyan, J.-P. Merlo, A. Mestvirishvili, A. Moeller, J. Nachtman, H. Ogul, Y. Onel, F. Ozok, A. Penzo, C. Snyder, E. Tiras, J. Wetzel, B. Blumenfeld, A. Cocoros, N. Eminizer, D. Fehling, L. Feng, A. V. Gritsan, W. T. Hung, P. Maksimovic, J. Roskes, M. Swartz, M. Xiao, C. Baldenegro Barrera, P. Baringer, A. Bean, S. Boren, J. Bowen, A. Bylinkin, T. Isidori, S. Khalil, J. King, G. Krintiras, A. Kropivnitskaya, C. Lindsey, D. Majumder, W. Mcbrayer, N. Minafra, M. Murray, C. Rogan, C. Royon, S. Sanders, E. Schmitz, J. D. Tapia Takaki, Q. Wang, J. Williams, G. Wilson, S. Duric, A. Ivanov, K. Kaadze, D. Kim, Y. Maravin, D. R. Mendis, T. Mitchell, A. Modak, A. Mohammadi, F. Rebassoo, D. Wright, A. Baden, O. Baron, A. Belloni, S. C. Eno, Y. Feng, N. J. Hadley, S. Jabeen, G. Y. Jeng, R. G. Kellogg, J. Kunkle, A. C. Mignerey, S. Nabili, F. Ricci-Tam, M. Seidel, Y. H. Shin, A. Skuja, S. C. Tonwar, K. Wong, D. Abercrombie, B. Allen, A. Baty, R. Bi, S. Brandt, W. Busza, I. A. Cali, M. D’Alfonso, G. Gomez Ceballos, M. Goncharov, P. Harris, D. Hsu, M. Hu, M. Klute, D. Kovalskyi, Y.-J. Lee, P. D. Luckey, B. Maier, A. C. Marini, C. Mcginn, C. Mironov, S. Narayanan, X. Niu, C. Paus, D. Rankin, C. Roland, G. Roland, Z. Shi, G. S. F. Stephans, K. Sumorok, K. Tatar, D. Velicanu, J. Wang, T. W. Wang, B. Wyslouch, A. C. Benvenuti, R. M. Chatterjee, A. Evans, S. Guts, P. Hansen, J. Hiltbrand, Sh. Jain, S. Kalafut, Y. Kubota, Z. Lesko, J. Mans, R. Rusack, M. A. Wadud, J. G. Acosta, S. Oliveros, K. Bloom, D. R. Claes, C. Fangmeier, L. Finco, F. Golf, R. Gonzalez Suarez, R. Kamalieddin, I. Kravchenko, J. E. Siado, G. R. Snow, B. Stieger, G. Agarwal, C. Harrington, I. Iashvili, A. Kharchilava, C. Mclean, D. Nguyen, A. Parker, J. Pekkanen, S. Rappoccio, B. Roozbahani, G. Alverson, E. Barberis, C. Freer, Y. Haddad, A. Hortiangtham, G. Madigan, D. M. Morse, T. Orimoto, L. Skinnari, A. Tishelman-Charny, T. Wamorkar, B. Wang, A. Wisecarver, D. Wood, S. Bhattacharya, J. Bueghly, T. Gunter, K. A. Hahn, N. Odell, M. H. Schmitt, K. Sung, M. Trovato, M. Velasco, R. Bucci, N. Dev, R. Goldouzian, M. Hildreth, K. Hurtado Anampa, C. Jessop, D. J. Karmgard, K. Lannon, W. Li, N. Loukas, N. Marinelli, I. Mcalister, F. Meng, C. Mueller, Y. Musienko, M. Planer, R. Ruchti, P. Siddireddy, G. Smith, S. Taroni, M. Wayne, A. Wightman, M. Wolf, A. Woodard, J. Alimena, B. Bylsma, L. S. Durkin, S. Flowers, B. Francis, C. Hill, W. Ji, A. Lefeld, T. Y. Ling, B. L. Winer, S. Cooperstein, G. Dezoort, P. Elmer, J. Hardenbrook, N. Haubrich, S. Higginbotham, A. Kalogeropoulos, S. Kwan, D. Lange, M. T. Lucchini, J. Luo, D. Marlow, K. Mei, I. Ojalvo, J. Olsen, C. Palmer, P. Piroué, J. Salfeld-Nebgen, D. Stickland, C. Tully, Z. Wang, S. Malik, S. Norberg, A. Barker, V. E. Barnes, S. Das, L. Gutay, M. Jones, A. W. Jung, A. Khatiwada, B. Mahakud, D. H. Miller, G. Negro, N. Neumeister, C. C. Peng, S. Piperov, H. Qiu, J. F. Schulte, J. Sun, F. Wang, R. Xiao, W. Xie, T. Cheng, J. Dolen, N. Parashar, K. M. Ecklund, S. Freed, F. J. M. Geurts, M. Kilpatrick, Arun Kumar, W. Li, B. P. Padley, R. Redjimi, J. Roberts, J. Rorie, W. Shi, A. G. Stahl Leiton, Z. Tu, A. Zhang, A. Bodek, P. de Barbaro, R. Demina, J. L. Dulemba, C. Fallon, T. Ferbel, M. Galanti, A. Garcia-Bellido, J. Han, O. Hindrichs, A. Khukhunaishvili, E. Ranken, P. Tan, R. Taus, B. Chiarito, J. P. Chou, A. Gandrakota, Y. Gershtein, E. Halkiadakis, A. Hart, M. Heindl, E. Hughes, S. Kaplan, S. Kyriacou, I. Laflotte, A. Lath, R. Montalvo, K. Nash, M. Osherson, H. Saka, S. Salur, S. Schnetzer, D. Sheffield, S. Somalwar, R. Stone, S. Thomas, P. Thomassen, H. Acharya, A. G. Delannoy, J. Heideman, G. Riley, S. Spanier, O. Bouhali, A. Celik, M. Dalchenko, M. De Mattia, A. Delgado, S. Dildick, R. Eusebi, J. Gilmore, T. Huang, T. Kamon, S. Luo, D. Marley, R. Mueller, D. Overton, L. Perniè, D. Rathjens, A. Safonov, N. Akchurin, J. Damgov, F. De Guio, S. Kunori, K. Lamichhane, S. W. Lee, T. Mengke, S. Muthumuni, T. Peltola, S. Undleeb, I. Volobouev, Z. Wang, A. Whitbeck, S. Greene, A. Gurrola, R. Janjam, W. Johns, C. Maguire, A. Melo, H. Ni, K. Padeken, F. Romeo, P. Sheldon, S. Tuo, J. Velkovska, M. Verweij, M. W. Arenton, P. Barria, B. Cox, G. Cummings, R. Hirosky, M. Joyce, A. Ledovskoy, C. Neu, B. Tannenwald, Y. Wang, E. Wolfe, F. Xia, R. Harr, P. E. Karchin, N. Poudyal, J. Sturdy, P. Thapa, S. Zaleski, J. Buchanan, C. Caillol, D. Carlsmith, S. Dasu, I. De Bruyn, L. Dodd, F. Fiori, C. Galloni, B. Gomber, M. Herndon, A. Hervé, U. Hussain, P. Klabbers, A. Lanaro, A. Loeliger, K. Long, R. Loveless, J. Madhusudanan Sreekala, T. Ruggles, A. Savin, V. Sharma, W. H. Smith, D. Teague, S. Trembath-reichert, N. Woods, CMS Collaboration

**Affiliations:** 1 Yerevan Physics Institute, Yerevan, Armenia; 2 Institut für Hochenergiephysik, Wien, Austria; 3 Institute for Nuclear Problems, Minsk, Belarus; 4 Universiteit Antwerpen, Antwerpen, Belgium; 5 Vrije Universiteit Brussel, Brussel, Belgium; 6 Université Libre de Bruxelles, Bruxelles, Belgium; 7 Ghent University, Ghent, Belgium; 8 Université Catholique de Louvain, Louvain-la-Neuve, Belgium; 9 Centro Brasileiro de Pesquisas Fisicas, Rio de Janeiro, Brazil; 10 Universidade do Estado do Rio de Janeiro, Rio de Janeiro, Brazil; 11 Universidade Estadual Paulista, Universidade Federal do ABC, São Paulo, Brazil; 12 Institute for Nuclear Research and Nuclear Energy, Bulgarian Academy of Sciences, Sofia, Bulgaria; 13 University of Sofia, Sofia, Bulgaria; 14 Beihang University, Beijing, China; 15 Department of Physics, Tsinghua University, Beijing, China; 16 Institute of High Energy Physics, Beijing, China; 17 State Key Laboratory of Nuclear Physics and Technology, Peking University, Beijing, China; 18 Universidad de Los Andes, Bogota, Colombia; 19 Universidad de Antioquia, Medellin, Colombia; 20 University of Split, Faculty of Electrical Engineering, Mechanical Engineering and Naval Architecture, Split, Croatia; 21 University of Split, Faculty of Science, Split, Croatia; 22 Institute Rudjer Boskovic, Zagreb, Croatia; 23 University of Cyprus, Nicosia, Cyprus; 24 Charles University, Prague, Czech Republic; 25 Escuela Politecnica Nacional, Quito, Ecuador; 26 Universidad San Francisco de Quito, Quito, Ecuador; 27 Academy of Scientific Research and Technology of the Arab Republic of Egypt, Egyptian Network of High Energy Physics, Cairo, Egypt; 28 National Institute of Chemical Physics and Biophysics, Tallinn, Estonia; 29 Department of Physics, University of Helsinki, Helsinki, Finland; 30 Helsinki Institute of Physics, Helsinki, Finland; 31 Lappeenranta University of Technology, Lappeenranta, Finland; 32 IRFU, CEA, Université Paris-Saclay, Gif-sur-Yvette, France; 33 Laboratoire Leprince-Ringuet, CNRS/IN2P3, Ecole Polytechnique, Institut Polytechnique de Paris, Paris, France; 34 Université de Strasbourg, CNRS, IPHC UMR 7178, Strasbourg, France; 35 Centre de Calcul de l’Institut National de Physique Nucleaire et de Physique des Particules, CNRS/IN2P3, Villeurbanne, France; 36 Université de Lyon, Université Claude Bernard Lyon 1, CNRS-IN2P3, Institut de Physique Nucléaire de Lyon, Villeurbanne, France; 37 Georgian Technical University, Tbilisi, Georgia; 38 Tbilisi State University, Tbilisi, Georgia; 39 RWTH Aachen University, I. Physikalisches Institut, Aachen, Germany; 40 RWTH Aachen University, III. Physikalisches Institut A, Aachen, Germany; 41 RWTH Aachen University, III. Physikalisches Institut B, Aachen, Germany; 42 Deutsches Elektronen-Synchrotron, Hamburg, Germany; 43 University of Hamburg, Hamburg, Germany; 44 Karlsruher Institut fuer Technologie, Karlsruhe, Germany; 45 Institute of Nuclear and Particle Physics (INPP), NCSR Demokritos, Aghia Paraskevi, Greece; 46 National and Kapodistrian University of Athens, Athens, Greece; 47 National Technical University of Athens, Athens, Greece; 48 University of Ioánnina, Ioánnina, Greece; 49 MTA-ELTE Lendület CMS Particle and Nuclear Physics Group, Eötvös Loránd University, Budapest, Hungary; 50 Wigner Research Centre for Physics, Budapest, Hungary; 51 Institute of Nuclear Research ATOMKI, Debrecen, Hungary; 52 Institute of Physics, University of Debrecen, Debrecen, Hungary; 53 Eszterhazy Karoly University, Karoly Robert Campus, Gyongyos, Hungary; 54 Indian Institute of Science (IISc), Bangalore, India; 55 National Institute of Science Education and Research, HBNI, Bhubaneswar, India; 56 Panjab University, Chandigarh, India; 57 University of Delhi, Delhi, India; 58 Saha Institute of Nuclear Physics, HBNI, Kolkata, India; 59 Indian Institute of Technology Madras, Chennai, India; 60 Bhabha Atomic Research Centre, Mumbai, India; 61 Tata Institute of Fundamental Research-A, Mumbai, India; 62 Tata Institute of Fundamental Research-B, Mumbai, India; 63 Indian Institute of Science Education and Research (IISER), Pune, India; 64 Institute for Research in Fundamental Sciences (IPM), Tehran, Iran; 65 University College Dublin, Dublin, Ireland; 66 INFN Sezione di Bari, Università di Bari, Politecnico di Bari, Bari, Italy; 67 INFN Sezione di Bologna, Università di Bologna, Bologna, Italy; 68 INFN Sezione di Catania, Università di Catania, Catania, Italy; 69 INFN Sezione di Firenze, Università di Firenze, Firenze, Italy; 70 INFN Laboratori Nazionali di Frascati, Frascati, Italy; 71 INFN Sezione di Genova, Università di Genova, Genoa, Italy; 72 INFN Sezione di Milano-Bicocca, Università di Milano-Bicocca, Milan, Italy; 73 INFN Sezione di Napoli, Università di Napoli ’Federico II’ , Napoli, Italy, Università della Basilicata, Potenza, Italy, Università G. Marconi, Rome, Italy; 74 INFN Sezione di Padova, Università di Padova, Padova, Italy, Università di Trento, Trento, Italy; 75 INFN Sezione di Pavia, Università di Pavia, Pavia, Italy; 76 INFN Sezione di Perugia, Università di Perugia, Perugia, Italy; 77 INFN Sezione di Pisa, Università di Pisa, Scuola Normale Superiore di Pisa, Pisa, Italy; 78 INFN Sezione di Roma, Sapienza Università di Roma, Rome, Italy; 79 INFN Sezione di Torino, Università di Torino, Torino, Italy, Università del Piemonte Orientale, Novara, Italy; 80 INFN Sezione di Trieste, Università di Trieste, Trieste, Italy; 81 Kyungpook National University, Daegu, Korea; 82 Chonnam National University, Institute for Universe and Elementary Particles, Kwangju, Korea; 83 Hanyang University, Seoul, Korea; 84 Korea University, Seoul, Korea; 85 Department of Physics, Kyung Hee University, Seoul, Korea; 86 Sejong University, Seoul, Korea; 87 Seoul National University, Seoul, Korea; 88 University of Seoul, Seoul, Korea; 89 Sungkyunkwan University, Suwon, Korea; 90 Riga Technical University, Riga, Latvia; 91 Vilnius University, Vilnius, Lithuania; 92 National Centre for Particle Physics, Universiti Malaya, Kuala Lumpur, Malaysia; 93 Universidad de Sonora (UNISON), Hermosillo, Mexico; 94 Centro de Investigacion y de Estudios Avanzados del IPN, Mexico City, Mexico; 95 Universidad Iberoamericana, Mexico City, Mexico; 96 Benemerita Universidad Autonoma de Puebla, Puebla, Mexico; 97 Universidad Autónoma de San Luis Potosí, San Luis Potosí, Mexico; 98 University of Montenegro, Podgorica, Montenegro; 99 University of Auckland, Auckland, New Zealand; 100 University of Canterbury, Christchurch, New Zealand; 101 National Centre for Physics, Quaid-I-Azam University, Islamabad, Pakistan; 102 AGH University of Science and Technology Faculty of Computer Science, Electronics and Telecommunications, Kraków, Poland; 103 National Centre for Nuclear Research, Swierk, Poland; 104 Institute of Experimental Physics, Faculty of Physics, University of Warsaw, Warsaw, Poland; 105 Laboratório de Instrumentação e Física Experimental de Partículas, Lisbon, Portugal; 106 Joint Institute for Nuclear Research, Dubna, Russia; 107 Petersburg Nuclear Physics Institute, Gatchina (St. Petersburg), Russia; 108 Institute for Nuclear Research, Moscow, Russia; 109 Institute for Theoretical and Experimental Physics named by A.I. Alikhanov of NRC ‘Kurchatov Institute’, Moscow, Russia; 110 Moscow Institute of Physics and Technology, Moscow, Russia; 111 National Research Nuclear University ’Moscow Engineering Physics Institute’ (MEPhI), Moscow, Russia; 112 P.N. Lebedev Physical Institute, Moscow, Russia; 113 Skobeltsyn Institute of Nuclear Physics, Lomonosov Moscow State University, Moscow, Russia; 114 Novosibirsk State University (NSU), Novosibirsk, Russia; 115 Institute for High Energy Physics of National Research Centre ‘Kurchatov Institute’, Protvino, Russia; 116 National Research Tomsk Polytechnic University, Tomsk, Russia; 117 Tomsk State University, Tomsk, Russia; 118 University of Belgrade: Faculty of Physics and VINCA Institute of Nuclear Sciences, Belgrade, Serbia; 119 Centro de Investigaciones Energéticas Medioambientales y Tecnológicas (CIEMAT), Madrid, Spain; 120 Universidad Autónoma de Madrid, Madrid, Spain; 121 Universidad de Oviedo, Instituto Universitario de Ciencias y Tecnologías Espaciales de Asturias (ICTEA), Oviedo, Spain; 122 Instituto de Física de Cantabria (IFCA), CSIC-Universidad de Cantabria, Santander, Spain; 123 University of Colombo, Colombo, Sri Lanka; 124 Department of Physics, University of Ruhuna, Matara, Sri Lanka; 125 CERN, European Organization for Nuclear Research, Geneva, Switzerland; 126 Paul Scherrer Institut, Villigen, Switzerland; 127 ETH Zurich-Institute for Particle Physics and Astrophysics (IPA), Zurich, Switzerland; 128 Universität Zürich, Zurich, Switzerland; 129 National Central University, Chung-Li, Taiwan; 130 National Taiwan University (NTU), Taipei, Taiwan; 131 Chulalongkorn University, Faculty of Science, Department of Physics, Bangkok, Thailand; 132 Çukurova University, Physics Department, Science and Art Faculty, Adana, Turkey; 133 Middle East Technical University, Physics Department, Ankara, Turkey; 134 Bogazici University, Istanbul, Turkey; 135 Istanbul Technical University, Istanbul, Turkey; 136 Istanbul University, Istanbul, Turkey; 137 Institute for Scintillation Materials of National Academy of Science of Ukraine, Kharkov, Ukraine; 138 National Scientific Center, Kharkov Institute of Physics and Technology, Kharkov, Ukraine; 139 University of Bristol, Bristol, UK; 140 Rutherford Appleton Laboratory, Didcot, UK; 141 Imperial College, London, UK; 142 Brunel University, Uxbridge, UK; 143 Baylor University, Waco, USA; 144 Catholic University of America, Washington, DC USA; 145 The University of Alabama, Tuscaloosa, USA; 146 Boston University, Boston, USA; 147 Brown University, Providence, USA; 148 University of California, Davis, Davis, USA; 149 University of California, Los Angeles, USA; 150 University of California, Riverside, Riverside, USA; 151 University of California, San Diego, La Jolla, USA; 152 University of California, Santa Barbara-Department of Physics, Santa Barbara, USA; 153 California Institute of Technology, Pasadena, USA; 154 Carnegie Mellon University, Pittsburgh, USA; 155 University of Colorado Boulder, Boulder, USA; 156 Cornell University, Ithaca, USA; 157 Fermi National Accelerator Laboratory, Batavia, USA; 158 University of Florida, Gainesville, USA; 159 Florida International University, Miami, USA; 160 Florida State University, Tallahassee, USA; 161 Florida Institute of Technology, Melbourne, USA; 162 University of Illinois at Chicago (UIC), Chicago, USA; 163 The University of Iowa, Iowa City, USA; 164 Johns Hopkins University, Baltimore, USA; 165 The University of Kansas, Lawrence, USA; 166 Kansas State University, Manhattan, USA; 167 Lawrence Livermore National Laboratory, Livermore, USA; 168 University of Maryland, College Park, USA; 169 Massachusetts Institute of Technology, Cambridge, USA; 170 University of Minnesota, Minneapolis, USA; 171 University of Mississippi, Oxford, USA; 172 University of Nebraska-Lincoln, Lincoln, USA; 173 State University of New York at Buffalo, Buffalo, USA; 174 Northeastern University, Boston, USA; 175 Northwestern University, Evanston, USA; 176 University of Notre Dame, Notre Dame, USA; 177 The Ohio State University, Columbus, USA; 178 Princeton University, Princeton, USA; 179 University of Puerto Rico, Mayaguez, USA; 180 Purdue University, West Lafayette, USA; 181 Purdue University Northwest, Hammond, USA; 182 Rice University, Houston, USA; 183 University of Rochester, Rochester, USA; 184 Rutgers, The State University of New Jersey, Piscataway, USA; 185 University of Tennessee, Knoxville, USA; 186 Texas A&M University, College Station, USA; 187 Texas Tech University, Lubbock, USA; 188 Vanderbilt University, Nashville, USA; 189 University of Virginia, Charlottesville, USA; 190 Wayne State University, Detroit, USA; 191 University of Wisconsin-Madison, Madison, WI USA; 192 CERN, 1211 Geneva 23, Switzerland

## Abstract

A measurement is presented of differential cross sections for *t*-channel single top quark and antiquark production in proton–proton collisions at a centre-of-mass energy of 13$\,\text {Te}\text {V}$ by the CMS experiment at the LHC. From a data set corresponding to an integrated luminosity of 35.9$\,\text {fb}^{-1}$, events containing one muon or electron and two or three jets are analysed. The cross section is measured as a function of the top quark transverse momentum ($p_{\mathrm{T}}$), rapidity, and polarisation angle, the charged lepton $p_{\mathrm{T}}$ and rapidity, and the $p_{\mathrm{T}}$ of the $\text {W}{}{}$  boson from the top quark decay. In addition, the charge ratio is measured differentially as a function of the top quark, charged lepton, and $\text {W}{}{}$  boson kinematic observables. The results are found to be in agreement with standard model predictions using various next-to-leading-order event generators and sets of parton distribution functions. Additionally, the spin asymmetry, sensitive to the top quark polarisation, is determined from the differential distribution of the polarisation angle at parton level to be $0.440 \pm 0.070$, in agreement with the standard model prediction.

## Introduction

**Fig. 1:**
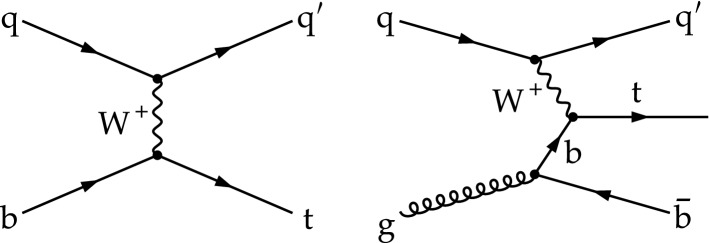
Born-level Feynman diagrams for single top quark production in the *t* channel. Corresponding diagrams also exist for single top antiquark production

The three main production modes of single top quarks and antiquarks in proton–proton (pp) collisions occur via electroweak interactions and are commonly categorised through the virtuality of the exchanged $\text {W}$  boson four-momentum. They are called *t* channel (*t* ch) when the four-momentum is space-like, *s* channel when it is time-like, and $\text {W}$-associated (${\text{tW}}$) when the four-momentum is on shell. At the CERN LHC, the production via the *t* channel has the largest cross section of the three modes whose most-relevant Born-level Feynman diagrams are shown in Fig. 1. In the rest of this paper, “quark” is used to generically denote a quark or an antiquark, unless otherwise specified.

The $t\text {-channel}$ production process was first observed by the D0 and CDF experiments at the Tevatron [1, 2]. Its inclusive cross section has been measured with high precision at the CERN LHC by the ATLAS and CMS Collaborations at $\sqrt{s}=7$, 8, and 13$\text {TeV}$  [3–8]. Differential cross sections have been determined as well at 7 and 8$\text {TeV}$  [3, 5, 9].

Differential cross section measurements can contribute to constraining the effective field theory operators [10], the top quark mass, the renormalisation and factorisation scales, and the parton distribution functions (PDFs) of the proton [11]. In particular, the ratio of the $t\text {-channel}$ top quark to antiquark production is sensitive to the ratio of the up to down quark content of the proton [12, 13]. Furthermore, differential angular distributions can be used to assess the electroweak coupling structure at the $\text {W}$tb vertex. A “vector−axial-vector” (V−A) coupling is predicted in the standard model (SM), leading to the production of highly polarised top quarks [14–16]. A powerful observable to investigate the coupling structure in $t\text {-channel}$ production is given by the top quark polarisation angle $\theta _{\mathrm{pol}}^\star$, defined via

1 $$\begin{aligned} \cos \theta _{\mathrm{pol}}^\star = \frac{\vec {p}_{{{\text {q}}}^{\prime }}^{\star }\cdot \vec {p}_{{\ell }{}{}}^{\star }}{|\vec {p}_{{{\text {q}}}^{\prime }}^{\star } | |\vec {p}_{{\ell }{}{}}^{\star } |}, \end{aligned}$$

where the superscript signifies that the momenta of the charged lepton, ${\ell }{}{}$ (muon or electron), from the top quark decay, and the spectator quark, ${{\text {q}}}^{\prime }$, are calculated in the top quark rest frame. The normalised differential cross section as a function of $\cos \theta _{\mathrm{pol}}^\star$ at the parton level is related to the top quark polarisation, *P*, as

2 $$\begin{aligned} \frac{1}{\sigma }\frac{\text {d}\sigma }{\text {d}\cos \theta _{\mathrm{pol}}^\star }=\frac{1}{2}\left( 1+2 A_{{\ell }{}{}}\cos \theta _{\mathrm{pol}}^\star \right) , A_{{\ell }{}{}}=\frac{1}{2}P\alpha _{{\ell }{}{}}, \end{aligned}$$

where $A_{{\ell }{}{}}$ denotes the spin asymmetry and $\alpha _{{\ell }{}{}}$ is the so-called spin-analysing power of the charged lepton [16]. The spin asymmetry and/or polarisation have been measured in ${\text {p}{}{}} {\text {p}{}{}}$ collision data by the ATLAS and CMS Collaborations at $\sqrt{s}=8$ $\,\text {Te}\text {V}$ using various analysis techniques [9, 17, 18].

In this paper, the differential cross section of combined single top quark and antiquark production in the *t* channel is measured by the CMS experiment at $\sqrt{s}=13$ $\,\text {Te}\text {V}$ as a function of the top quark transverse momentum ($p_{\mathrm{T}}$), rapidity, and polarisation angle, the $p_{\mathrm{T}}$ and rapidity of the charged lepton that originates from the top quark decay, and the $p_{\mathrm{T}}$ of the $\text {W}{}{}$  boson from the top quark decay. The spin asymmetry is further determined from the measured differential cross section with respect to the polarisation angle. Additionally, a measurement of the differential charge ratio is performed as a function of the $p_{\mathrm{T}}$ and rapidities of the top quark and charged lepton, and the $p_{\mathrm{T}}$ of the $\text {W}{}{}$  boson. Differential cross sections are measured at both the parton and particle levels using an unfolding procedure.

The analysis strategy and the structure of the paper are outlined in the following. A brief description of the CMS detector is given in Sect. 2, followed by a summary of the analysed data and simulated event samples in Sect. 3. The reconstruction of physics objects and the event selection are detailed in Sect. 4. To determine the contributions from signal and backgrounds a maximum-likelihood fit (ML) is performed separately in each bin of the measurement. In the fit, shape distributions, referred to in the following as templates, are fitted to the data. For the signal and all background processes, samples of simulated events are used to determine the shape distributions, except for the templates of events containing only jets produced through the strong interaction, which are referred to as “multijet” events in this paper. The procedure to estimate the templates of multijet events based on data in a sideband region is provided in Sect. 5. Section 6 describes the measurement of the number of $t\text {-channel}$ single top quark events from data through an ML fit. In the fit, statistical and experimental systematic uncertainties are profiled, where the latter encompasses uncertainties related to the reconstruction, identification, and calibration of the selected events and physics objects. The resulting distributions of the observables are validated in control and signal regions in Sect. 7. The fit results are input to an unfolding procedure to determine the differential cross sections and charge ratios at the parton and particle levels, as detailed in Sect. 8. The sources of experimental and theoretical systematic uncertainties are described in Sect. 9. The results are presented in Sect. 10 and the paper is summarised in Sect. 11.

## The CMS detector and event reconstruction

The central feature of the CMS apparatus is a superconducting solenoid of 6$\,\text {m}$ internal diameter, providing a magnetic field of 3.8$\,\text {T}$. Within the solenoid volume are a silicon pixel and strip tracker, a lead tungstate crystal electromagnetic calorimeter (ECAL), and a brass and scintillator hadron calorimeter (HCAL), each composed of a barrel and two endcap sections. Forward calorimeters (HF) extend the pseudorapidity ($\eta$) coverage provided by the barrel and endcap detectors. Muons are detected in gas-ionisation chambers embedded in the steel flux-return yoke outside the solenoid. A more detailed description of the CMS detector, together with a definition of the coordinate system used and the relevant kinematic variables, can be found in Ref. [19].

The particle-flow (PF) algorithm [20] aims to reconstruct and identify each particle in an event with an optimised combination of information from various elements of the CMS detector. The energy of electrons is estimated from a combination of the electron momentum at the primary interaction vertex, as determined by the tracker, the energy of the corresponding ECAL cluster, and the energy sum of all bremsstrahlung photons spatially compatible with originating from the electron track. The energy of muons is obtained from the curvature of a global track estimated from reconstructed hits in the inner tracker and muon systems. The energy of charged hadrons is determined from a combination of their momentum measured in the tracker and the matching ECAL and HCAL energy deposits. Finally, the energy of neutral hadrons is obtained from the corresponding ECAL and HCAL energy deposits. In the regions $|\eta |>3$, electromagnetic and hadronic shower components are identified in the HF.

Events of interest are selected using a two-tiered trigger system [21]. The first level, composed of custom hardware processors, uses information from the calorimeters and muon detectors whereas a version of the full event reconstruction software optimised for fast processing is performed at the second level, which runs on a farm of processors.

The missing transverse momentum vector, ${\vec p}_{\mathrm{T}}^{\text {miss}}$, is defined as the projection onto the plane perpendicular to the beams of the negative vector momentum sum of all PF candidates in an event. Its magnitude is referred to as $p_{\mathrm{T}} ^\text {miss}$.

## Data set and simulated samples

The analysed ${\text {p}{}{}} {\text {p}{}{}}$ collision data set was recorded in 2016 by the CMS detector and corresponds to an integrated luminosity of 35.9$\,\text {fb}^{-1}$  [22]. Events were triggered by requiring at least one isolated muon candidate with $p_{\mathrm{T}} >24$ $\,\text {Ge}\text {V}$ and $|\eta | < 2.4$ or one electron candidate with $p_{\mathrm{T}} >32$ $\,\text {Ge}\text {V}$ and $|\eta | < 2.1$, with additional requirements [23] that select genuine electrons with an efficiency of about 80%.

Various samples of simulated events are used in this measurement to evaluate the detector resolution, efficiency, and acceptance, estimate the contributions from background processes, and determine the differential cross sections at the parton and particle levels.

Single top quark events in the *t* channel are simulated at next-to-leading order (NLO) in the four-flavour scheme (4FS) with powheg  v2 [24, 25] interfaced with pythia v8.212 [26] for the parton shower simulation, using the CUETP8M1 [27] tune interfaced with madspin  [28] for simulating the top quark decay. For comparison, alternative NLO $t\text {-channel}$ samples have been generated in the 4FS and five-flavour scheme (5FS), using MadGraph 5_amc@nlo v2.2.2 [29] interfaced with pythia.

The powheg  v2 generator is also used to simulate events from top quark pair production ($\mathrm {t}\bar{\mathrm {t}}$) at NLO. Parton showering is simulated with pythia using the CUETP8M2T4 tune [30]. The production of single top quark events via the tW channel is simulated at NLO using powheg  v1 [31] in the 5FS interfaced with pythia using the CUETP8M1 tune for the parton shower simulation. The overlap with top quark pair production is removed by applying the diagram removal scheme [32]. Samples of $\text {W}{}{} \text {+jets}$ events are generated with MadGraph 5_amc@nlo v2.3.3 at NLO, and interfaced with pythia using the CUETP8M1 tune. The production of leptonically decaying $\text {W}{}{}$  bosons in association with jets is simulated with up to two additional partons at the matrix element level, and the FxFx scheme [33] is used for jet merging. Lastly, $\text {Z}{}{}/{{\upgamma }{}{}} ^{*}\text {+jets}$ events are generated with MadGraph 5_amc@nlo v2.2.2 at leading order (LO), interfaced with pythia using the MLM jet matching scheme [34].

In these simulated samples, the NNPDF3.0 [35] NLO set is used as the default PDF, and a nominal top quark mass of 172.5$\,\text {Ge}\text {V}$ is chosen where applicable. The simulated events are overlaid with additional collision interactions (“pileup”) according to the distribution inferred from the data. All generated events undergo a full Geant4  [36] simulation of the detector response.

The $t\text {-channel}$ cross section in ${\text {p}{}{}} {\text {p}{}{}}$ collisions at $\sqrt{s}=13$ $\,\text {Te}\text {V}$ is predicted to be $\sigma _{{\text {t}}} =136.0{\,}^{+5.4}_{-4.6}$ $\,\text {pb}$ for the top quark and $\sigma _{{ {{\text {t}}}}} =81.0{\,}^{+4.1}_{-3.6}$ $\,\text {pb}$ for the top antiquark, calculated for a top quark mass of 172.5$\,\text {Ge}\text {V}$ at NLO in quantum chromodynamics (QCD) using the hathor v2.1 [11, 37] program. The PDF and the strong coupling constant ($\alpha _S$) uncertainties are calculated using the PDF4LHC prescription [38, 39] with the MSTW2008 NLO 68% confidence level [40, 41], CT10 [42] NLO, and NNPDF2.3 [43] NLO PDF sets, and are added in quadrature with the renormalisation and factorisation scale uncertainty. The simulated samples of single top quark and antiquark events employed in this measurement—generated with similar settings—were normalised using the predicted cross sections above. Predictions at next-to-next-to-leading order are available as well [12] and are 3% smaller than the corresponding cross sections at NLO. However, these are not utilised since they have been calculated using a different PDF set and top quark mass value.

## Event selection

Proton–proton collision events containing one isolated muon or electron and two or three jets are analysed. This signature selects events where the $\text {W}{}{}$  boson from a single top quark decays into a charged lepton and a neutrino. One of the selected jets is expected to stem from the hadronisation of a bottom quark that originates from the top quark decay. Another jet ($j^{\prime }$) from a light-flavoured quark (up, down, or strange) is expected from the spectator quark (labelled ${{\text {q}}}^{\prime }$ in Fig. 1) that is produced in association with the top quark. The jet from the spectator quark is characteristically found at relatively low angles with respect to the beam axis.

The reconstructed vertex with the largest value of summed physics-object $p_{\mathrm{T}} ^2$ is taken to be the primary ${\text {p}{}{}} {\text {p}{}{}}$ interaction vertex. The physics objects are the jets, clustered using the jet finding algorithm described in Refs. [44, 45] with the tracks assigned to the vertex as inputs, and the negative vector $\vec {p}_\mathrm {T}$ sum of those jets.

Muon candidates are accepted if they have $p_{\mathrm{T}} >26$ $\,\text {Ge}\text {V}$, $|\eta |<2.4$, and pass the following identification requirements optimised for the selection of genuine muons. A global muon track must have a track fit with a $\chi ^2$ per degree of freedom <10, have hits in the silicon tracker and muon systems, including at least six in the tracker, of which at least one must be in the pixel detector. Additionally, track segments are required in at least two muon stations to suppress signals from hadronic showers spilling into the muon system. Muon candidates are required to be isolated with a relative isolation parameter $I_{\mathrm{rel}}^{{{\upmu }{}{}}} <6$%, which is defined as the scalar sum of the transverse energies $E_{\mathrm{T}}$ deposited in the ECAL and HCAL within a cone of radius $\varDelta R = \sqrt{\smash [b]{(\varDelta \eta )^2+(\varDelta \phi )^2}} < 0.4$, divided by the muon $p_{\mathrm{T}}$. The transverse energy is defined as $E_{\mathrm{T}} =E\,\sin (\theta )$ with *E* and $\theta$ being the energy and polar angle, respectively, of photons and charged and neutral hadrons. Here, $\varDelta \eta$ and $\varDelta \phi$ are the pseudorapidity and azimuthal angle, respectively, measured relative to the muon direction. The isolation parameter is corrected by subtracting the energy deposited by pileup, which is estimated from the energy deposited by charged hadrons within the isolation cone that are associated with pileup vertices [46].

Electron candidates are required to have $p_{\mathrm{T}} >35$ $\,\text {Ge}\text {V}$, $|\eta |<1.48$, and fulfil a set of additional quality requirements as follows: the distance between the matched ECAL cluster position and the extrapolated electron track has to be within $|\varDelta \eta |<3.08\times 10^{-3}$ and $|\varDelta \phi |<8.16\times 10^{-2}$; the absolute difference between the inverse of the energy estimated from the ECAL cluster and the inverse of the electron track momentum must be less than $12.9\,\text {Me}\text {V} ^{-1}$; the ratio of the HCAL to the ECAL energy associated with the electron is required to be less than 4.14%; the energy-weighted lateral width of the electron shower in the ECAL along the $\eta$ direction is restricted to ${<}9.98\times 10^{-3}$. Electrons from photon conversions are suppressed by requiring that the corresponding track has no missing hits in the inner layers of the tracker and that they do not stem from a photon conversion vertex. Electron candidates have to be isolated using the so-called effective-area-corrected relative isolation parameter [47] by requiring $I_\text {rel}^{{\text {e}}} <5.88$%. This parameter is defined similarly to the muon isolation parameter as the sum of the charged and neutral particle energies within a cone of $\varDelta R<0.3$ around the electron candidate, divided by the electron $p_{\mathrm{T}}$. The relative contribution from pileup is estimated as $A_\text {eff}\,\rho$ and subtracted from the isolation parameter, where $A_\text {eff}$ denotes an $\eta$-dependent effective area, and $\rho$ is the median of the $E_{\mathrm{T}}$ density in a $\delta \eta {\times }\delta \phi$ region calculated using the charged particle tracks associated with the pileup vertices.

The selected muon (electron) candidate has to be within 2.0 (0.5)$\,\text {mm}$ in the transverse plane and 5.0 (1.0)$\,\text {mm}$ along the beam direction of the primary vertex.

Electron candidates with showers in the ECAL endcap ($1.48<|\eta |<2.5$) are not used in the measurement because of the higher background consisting of hadrons misidentified as electrons and of electrons originating from decays of heavy-flavour hadrons, which is found to be about four times larger compared to the ECAL barrel region.

Events are rejected if additional muon or electron candidates passing looser selection criteria are present. The selection requirements for these additional muons/electrons are as follows: looser identification and isolation criteria, $p_{\mathrm{T}} >10$ (15)$\,\text {Ge}\text {V}$ for muons (electrons), and $|\eta |<2.5$.

The transverse $\text {W}{}{}$  boson mass is calculated from the formula

3 $$\begin{aligned} m_{\mathrm{T}} (\text {W}{}{}) =\sqrt{\smash [b]{2p_{\mathrm{T}} ^{\ell }p_{\mathrm{T}} ^\text {miss} \left[ 1-\cos (\phi ^\ell -\phi ^\text {miss})\right] }} \end{aligned}$$

using the $p_{\mathrm{T}}$ and the $\phi$ of the charged lepton and ${\vec p}_{\mathrm{T}}^{\text {miss}}$.

Jets are reconstructed from PF candidates and clustered by applying the anti-$k_{\mathrm{T}}$ algorithm [44] with a distance parameter of 0.4 using the FastJet package [45]. The influence of pileup is mitigated using the charged hadron subtraction technique [48]. The jet momentum is determined as the vectorial sum of all particle momenta in the jet. An offset correction is applied to the jet $p_{\mathrm{T}}$ to account for contributions from pileup. Further corrections are applied to account for the nonuniform detector response in $\eta$ and $p_{\mathrm{T}}$ of the jets. The corrected jet momentum is found from simulation to be within 2 to 10% of the true momentum over the whole $p_{\mathrm{T}}$ spectrum and detector acceptance. The corrections are propagated to the measured ${\vec p}_{\mathrm{T}}^{\text {miss}}$. A potential overlap of a jet with the selected lepton is removed by ignoring jets that are found within a cone of $\varDelta R<0.4$ around a selected lepton candidate. The analysis considers jets within $|\eta |<4.7$ whose calibrated $p_{\mathrm{T}}$ is greater than 40$\,\text {Ge}\text {V}$, with the exception of the HCAL–HF transition region ($2.7<|\eta |<3$) in which jets must have a $p_{\mathrm{T}}$ of at least 50$\,\text {Ge}\text {V}$ to reduce the contribution from detector noise. The event is accepted for further analysis if two or three jets are present.

To reduce the large background from $\text {W}{}{} \text {+jets}$ events, a b  tagging algorithm based on a multivariate analysis (MVA) called “combined MVA” [49], which combines the results from various other b  tagging algorithms, is used for identifying jets produced from the hadronisation of b  quarks within the acceptance of the silicon tracker ($|\eta |<2.4$). A tight selection is applied on the discriminant of the algorithm, which gives an efficiency of ${\approx }$50% for jets originating from true b  quarks and misidentification rates of ${\approx }$0.1% for light jets from u, d, or s quarks or gluons and ${\approx }$3% for jets from c quarks, as determined from simulation.

Corrections are applied to the simulated events to account for known differences with respect to data. Lepton trigger, reconstruction, and identification efficiencies are estimated with a “tag-and-probe” method [50] from $\text {Z}{}{}/{{\upgamma }{}{}} ^{*}\text {+jets}$ events for data and simulation from which corrections are derived in bins of lepton $\eta$ and $p_{\mathrm{T}}$. The b  tagging performance in simulation is corrected to match the tagging efficiency observed in data, using scale factors that depend on the $p_{\mathrm{T}}$ and $\eta$ of the selected jets. The scale factors are estimated by dedicated analyses performed with independent data samples [49]. In particular, the mistagging rate of non-b jets in data is determined using the “negative-tag” method [51]. A smearing of the jet momenta is applied to account for the known difference in jet energy resolution in simulation compared to data. The profile of pileup interactions is reweighted in simulation to match the one in data derived from the measured instantaneous luminosity.

To classify signal and control samples of events, different event categories are defined, denoted “*N*j*M*b ”, where *N* is the total number of selected jets (2 or 3) and *M* is the number of those jets passing the b  tagging requirement (0, 1, or 2). The 2j1b category has the highest sensitivity to the signal yield, whereas the 2j0b and 3j2b categories, enriched in background processes with different compositions, are used to assess the background modelling.

One top quark candidate is reconstructed per event in the 2j1b signal category assuming $t\text {-channel}$ single top quark production. The procedure commences by first reconstructing the $\text {W}{}{}$  boson. The component of the neutrino candidate momentum along the beam direction $p_{z}$ is found by imposing a $\text {W}{}{}$  boson mass constraint (80.4$\,\text {Ge}\text {V}$) on the system formed by the charged lepton and ${\vec p}_{\mathrm{T}}^{\text {miss}}$, the latter being interpreted as the projection in the transverse plane of the four-momentum of the unknown neutrino, as in Ref. [52]. The four-momentum of the top quark candidate (from which its mass, $p_{\mathrm{T}}$, and rapidity are derived) is then calculated as the vector sum of the four-momenta of the charged lepton, the b-tagged jet, and the neutrino candidate. The other (nontagged) jet is interpreted as originating from the spectator quark, which recoils against the $\text {W}{}{}$  boson.

## Multijet background estimation

Since the probability for a simulated multijet event to mimic the final state of the signal process is very small, it becomes impractical to simulate a sufficiently large number of events for this background. Therefore, the background from multijet events in the analysis phase space region is estimated in a two-step procedure based on data in a sideband region. First, templates of the $m_{\mathrm{T}} (\text {W}{}{})$ distribution from multijet events are obtained from data in a sideband region. Their normalisations are then estimated in a second step through a template-based ML fit to the events in the 2j1b and 3j2b categories, simultaneously with the number of signal events, as described in Sect. 6. In this section, a dedicated ML fit is discussed that is performed on events in the 2j0b category only for validating the procedure. The outcome of this ML fit is not used further in the measurement.

In the muon channel, the sideband region is defined by inverting the muon isolation requirement ($I_{\mathrm{rel}}^{{{\upmu }{}{}}} >20\%$), which results in a region dominated by multijet events. In the electron channel, the electron candidate is required to fail loose identification criteria, yielding a sideband region consisting not only of nonisolated electrons but also of electrons that fail the photon conversion criteria or are accompanied by large amounts of bremsstrahlung, thus reflecting a combination of various effects. The templates used in the ML fit are determined for this category by subtracting the contamination from other processes, estimated using simulation and which amounts to about 10 (5)% in the muon (electron) channel, from the data.

**Fig. 2:**
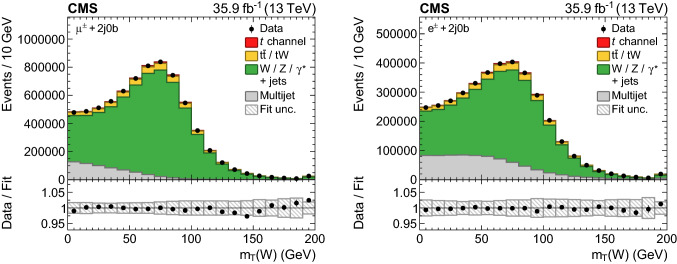
Distributions of the transverse $\text {W}{}{}$  boson mass in the 2 jets, 0 b  tag control category for the (left) muon and (right) electron channels after scaling the simulated and multijet templates to the result of a dedicated ML fit performed on this category of events. The hatched band displays the fit uncertainty. The lower plots give the ratio of the data to the fit results. The right-most bins include the event overflows

The template shapes have been validated for various observables in the 2j0b $\text {W}{}{} \text {+jets}$ control category where the fraction of selected multijet events amounts to approximately 10 (20)% for muon (electron) events, which is comparable to those in the signal category. The $m_{\mathrm{T}} (\text {W}{}{})$ distributions are shown in Fig. 2 for the muon (left) and electron (right) channel after the multijet templates (extracted from data) and the templates of the processes with prompt leptons (extracted from the simulated events) have been normalised to the result of a dedicated ML fit using only events in the 2j0b category. This dedicated fit encompasses only two components, which are the multijet template whose yield is unconstrained in the fit, and all other processes grouped together, with a constraint of ${\pm }30\%$ on their combined yield using a log-normal prior. The fit is performed while simultaneously profiling the impact of experimental systematic uncertainties (as discussed in Sect. 9) affecting the yield and shape of the templates. After the fit, the derived multijet templates and the simulated samples in both channels are found to describe the distributions of data well, thus validating the procedure for estimating the contribution of multijet events from data. For the measurement, the normalisations of the multijet templates in the 2j1b and 3j2b categories are estimated using a different procedure, as described in Sect. 6.

## Signal yield estimation

The number of $t\text {-channel}$ single top quark events in data is determined from an ML fit using the distributions of $m_{\mathrm{T}} (\text {W}{}{})$ and of two boosted decision tree (BDT) discriminants in the 2j1b category, and the $m_{\mathrm{T}} (\text {W}{}{})$ distribution in the 3j2b category. Simultaneously, the background yields and the impact of the experimental systematic uncertainties, modelled using nuisance parameters that influence yield and shape, are profiled.

The first BDT, labelled $\mathrm {BDT}_{t\text {-ch}}$, has been trained separately on muon and electron events to discriminate $t\text {-channel}$ single top quark events from $\mathrm {t}\bar{\mathrm {t}}$, $\text {W}{}{} \text {+jets}$, and multijet events using corresponding samples of simulated events. The following five observables have been chosen as input:

- the absolute value of the pseudorapidity of the untagged jet, $|\eta (j^{\prime }) |$;
- the reconstructed top quark mass, $m_{{\ell }{}{} {{\upnu }{}{}} {{\text {b}}}}$;
- the transverse $\text {W}{}{}$  boson mass, $m_{\mathrm{T}} (\text {W}{}{})$;
- the distance in $\eta \text {--}\phi$ space ($\varDelta R$) between the b-tagged and the untagged jet, $\varDelta R({\text {b}},j^{\prime })$;
- the absolute difference in pseudorapidity between the b-tagged jet used to reconstruct the top quark and the selected lepton, $|\varDelta \eta ({\text {b}},{\ell }{}{})|$.

**Fig. 3:**
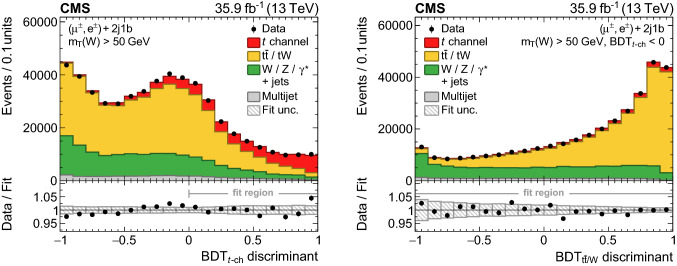
Distributions of the BDT discriminants in the 2 jets, 1 b  tag category: (left) $\mathrm {BDT}_{t\text {-ch}}$ trained to separate signal from background events; (right) $\mathrm {BDT}_{\mathrm {t}\bar{\mathrm {t}}/\text {W}{}{}}$ trained to separate $\mathrm {t}\bar{\mathrm {t}}$ from $\text {W}{}{} \text {+jets}$ events in a background-dominated category. Events in the muon and electron channels have been summed. The predictions have been scaled to the result of the inclusive ML fit and the hatched band displays the fit uncertainty. The regions of the distributions used in the fits are indicated in the lower panels, which show the ratio of the data to the fit result

These have been selected based on their sensitivity for separating signal from background events, while exhibiting low correlations with the observables used to measure the differential cross sections. The resulting distribution of the $\mathrm {BDT}_{t\text {-ch}}$ discriminant is presented in Fig. 3 (left).

The $\mathrm {BDT}_{t\text {-ch}}$ discriminant shapes of the $\text {W}{}{} \text {+jets}$ and $\mathrm {t}\bar{\mathrm {t}}$ backgrounds are found to be very similar. To obtain sensitivity in the fit to both backgrounds individually, a second BDT, labelled $\mathrm {BDT}_{\mathrm {t}\bar{\mathrm {t}}/\text {W}{}{}}$, has been trained separately on muon and electron events to classify events only for these two processes using the following six input observables: $m_{{\ell }{}{} {{\upnu }{}{}} {{\text {b}}}}$; $p_{\mathrm{T}} ^\text {miss}$; $\varDelta R({\text {b}},j^{\prime })$; $|\varDelta \eta ({\text {b}},{\ell }{}{})|$; the $\text {W}{}{}$  boson helicity angle, $\cos \theta _{\text {W}{}{}}^\star$, defined as the angle between the lepton momentum and the negative of the top quark momentum in the $\text {W}{}{}$  boson rest frame [16]; and the event shape *C*, defined using the momentum tensor

4 $$\begin{aligned} S^{ab}=\frac{\sum _{i}^{\text {jets},{\ell }{}{},{\vec p}_{\mathrm{T}}^{\text {miss}}}p_{i}^{a} p_{i}^{b}}{\sum _{i}^{\text {jets},{\ell }{}{},{\vec p}_{\mathrm{T}}^{\text {miss}}}|\vec {p}_{i} |^2}, \end{aligned}$$

as $C=3(\lambda _1\lambda _2+\lambda _1\lambda _3+\lambda _2\lambda _3)$, where $\lambda _{1}$, $\lambda _{2}$, and $\lambda _{3}$ denote the eigenvalues of the momentum tensor $S^{ab}$ with $\lambda _1+\lambda _2+\lambda _3=1$. In the two most extreme cases, the event shape *C* vanishes for perfectly back-to-back dijet events ($C=0$) and reaches its maximum ($C=1$) if the final-state momenta are distributed isotropically. For the measurement, the $\mathrm {BDT}_{\mathrm {t}\bar{\mathrm {t}}/\text {W}{}{}}$ discriminant is evaluated only in the phase space region defined by $m_{\mathrm{T}} (\text {W}{}{}) >50$ $\,\text {Ge}\text {V}$ and $\mathrm {BDT}_{t\text {-ch}} <0$, which is found to be largely dominated by background events. Thus, the $\mathrm {BDT}_{\mathrm {t}\bar{\mathrm {t}}/\text {W}{}{}}$ input observables do not have to be selected explicitly such that they possess low correlation with the observables used to measure the differential cross sections. The resulting $\mathrm {BDT}_{\mathrm {t}\bar{\mathrm {t}}/\text {W}{}{}}$ discriminant distribution is displayed in Fig. 3 (right).

The ML fit is performed using the following four distributions from events in various categories:

- the $m_{\mathrm{T}} (\text {W}{}{})$ distribution for events with $m_{\mathrm{T}} (\text {W}{}{}) <50$ $\,\text {Ge}\text {V}$ in the 2j1b category, which is particularly sensitive to the number of multijet events;
- the $\mathrm {BDT}_{\mathrm {t}\bar{\mathrm {t}}/\text {W}{}{}}$ discriminant distribution for events with $m_{\mathrm{T}} (\text {W}{}{}) >50$ $\,\text {Ge}\text {V}$ and $\mathrm {BDT}_{t\text {-ch}} <0$ in the 2j1b category, which defines a region enriched in $\mathrm {t}\bar{\mathrm {t}}$ and $\text {W}{}{} \text {+jets}$ but depleted of signal and multijet events;
- the $\mathrm {BDT}_{t\text {-ch}}$ discriminant distribution for events with $m_{\mathrm{T}} (\text {W}{}{}) >50$ $\,\text {Ge}\text {V}$ and $\mathrm {BDT}_{t\text {-ch}} >0$ in the 2j1b category, which is enriched in signal events;
- the $m_{\mathrm{T}} (\text {W}{}{})$ distribution in the 3j2b category, which provides additional sensitivity to the $\mathrm {t}\bar{\mathrm {t}}$ yield, and thus further reduces the correlation between the estimated yields.

**Fig. 4:**
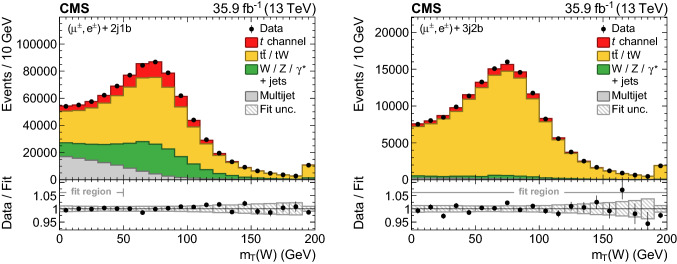
Distributions of the transverse $\text {W}{}{}$  boson mass for events in the (left) 2 jets, 1 b  tag and (right) 3 jets, 2 b  tags categories. Events in the muon and electron channels have been summed. The predictions have been scaled to the result of the inclusive ML fit and the hatched band displays the fit uncertainty. The regions of the distributions used in the fits are indicated in the lower panels, which show the ratio of the data to the fit result. The right-most bins include the event overflows

The $m_{\mathrm{T}} (\text {W}{}{})$ distributions in the 2j1b and 3j2b categories are shown in Fig. 4 on the left and right, respectively. In the fit, each distribution is split in two by separating events depending on the charge of the selected muon or electron in the event. This results in eight distributions per lepton channel and thus 16 distributions in the ${{\upmu }{}{}}/{\text {e}}$ combined fit. A coarser equidistant binning of the distributions, as opposed to the one shown in Figs. 3 and 4, is used in the ML fits to prevent cases where single bins are depleted of background estimates as follows: four bins are used for each of the $m_{\mathrm{T}} (\text {W}{}{})$ and $\mathrm {BDT}_{t\text {-ch}}$ distributions in the 2j1b category; eight bins are used for the $\mathrm {BDT}_{\mathrm {t}\bar{\mathrm {t}}/\text {W}{}{}}$ distribution; and ten bins are used for the $m_{\mathrm{T}} (\text {W}{}{})$ distribution in the 3j2b category.

The yields of $t\text {-channel}$ single top quark and antiquark events are measured independently. Background events containing top quarks ($\mathrm {t}\bar{\mathrm {t}}$, ${\text{tW}}$) are grouped together, and only their total yield is estimated. The top quark background yield is constrained using a log-normal prior with a width of ${\pm }10$% to account for the uncertainty in the theoretical $\mathrm {t}\bar{\mathrm {t}}$ and ${\text{tW}}$ production cross sections, and the uncertainty when two out of the four jets expected from semileptonic $\mathrm {t}\bar{\mathrm {t}}$ production are not within the acceptance, as is the case in the 2j1b category. The electroweak background processes, $\text {W}{}{} \text {+jets}$ and $\text {Z}{}{}/{{\upgamma }{}{}} ^{*}\text {+jets}$, are grouped together as well, and an uncertainty of ${\pm }30$% in their combined yield is applied using a log-normal prior constraint. This is motivated by the theoretical uncertainty in the modelling of the $\text {W}{}{}$ and $\text {Z}{}{}/{{\upgamma }{}{}} ^{*}$ production rates in association with two or more (heavy-flavour) jets [53, 54]. The yields of multijet events are assumed to be independent per lepton type and event category. Their yields are constrained by a log-normal prior with a width of ${\pm }100$% with respect to the template normalisations obtained from data in the sideband regions. In addition, an uncertainty in the predicted lepton charge ratio per background process, accounting for charge misreconstruction and uncertainties in the charge ratio [55], is taken into account using a Gaussian prior with a width of ${\pm }1$% in the fit, for a total of 14 fit parameters. The impact of the finite number of simulated events on the templates is accounted for by employing the “Barlow–Beeston-lite” method [56].

Experimental systematic uncertainties, as detailed in Sect. 9, are profiled in the fit simultaneously with the yields and charge ratios. Each source is assigned a nuisance parameter according to which the shape and yield of the fit templates are modified.

The resulting event yields from a simultaneous fit to the data in the muon and electron channels are listed in Table 1. Overall, the distributions used in the fit, shown in Figs. 3 and 4, are found to be well modelled by the samples of simulated events and the multijet templates from data after normalising them to the fit result.

**Table 1:** Measured and observed event yields in the 2j1b category for each lepton channel and charge. The uncertainties in the yields are the combination of statistical and experimental systematic uncertainties

Process	${\mu}^{+}$	${\mu}^{-}$	$\hbox {e}^{+}$	$\hbox {e}^{-}$
$\text {W}{}{}/\text {Z}{}{}/{{\upgamma }{}{}} ^{*}\text {+jets}$	$72\,000 \pm 6800$	$62\,800 \pm 5600$	$33\,400 \pm 3200$	$30\,700 \pm 2800$
$\mathrm {t}\bar{\mathrm {t}}$/${\text{tW}}$	$142\,400 \pm 2400$	$143\,400 \pm 2500$	$84\,500 \pm 1400$	$84\,800 \pm 1500$
Multijet	$35\,150 \pm 550$	$35\,710 \pm 760$	$13\,500 \pm 1000$	$12\,700 \pm 1000$
*t* channel (top quark)	$34\,400 \pm 1500$	$10 \pm 3$	$17\,720 \pm 820$	$27 \pm 2$
*t* channel (top antiquark)	$13 \pm 2$	$21\,600 \pm 1600$	$25 \pm 3$	$11\,460 \pm 880$
Total	$284\,100 \pm 5800$	$263\,700 \pm 4600$	$149\,300 \pm 2400$	$139\,700 \pm 2200$
Data	283 391	260 044	148 418	138 781

For each differential cross section measurement, the observable of interest is divided into intervals, discussed in Sect. 8, and a fit is performed in which the signal and background yields can vary independently in each of the intervals. The likelihood*L* to be maximised in such fits can be expressed as

5 $$\begin{aligned}&\ln \left( L(\vec {\beta },\vec {\nu },\vec {R})\right) \nonumber \\&\quad = -\sum _{k}^\text {dist}\sum _{j}^\text {int}\sum _{i}^\text {bins}\left( d_{kji}\ln {p_{kji}(\vec {\beta _{j}},\vec {\nu },\vec {R})-p_{kji}(\vec {\beta _{j}},\vec {\nu },\vec {R})}\right) \nonumber \\&\qquad +\text {constraints}, \end{aligned}$$

where *d* denotes the number of observed events and *p* is the estimated yield. The summation over*k* denotes the 16 distributions (“dist”), *j* denotes the interval (“int”) in the observable (e.g. for the top quark $p_{\mathrm{T}}$: 0–50$\,\text {Ge}\text {V}$, 50–80 $\,\text {Ge}\text {V}$, 80–120$\,\text {Ge}\text {V}$, 120–180 $\,\text {Ge}\text {V}$, and 180–300$\,\text {Ge}\text {V}$), and *i* denotes a bin in one of the 16 distributions per interval. The prediction $\vec {p}_{kj}$, which includes all bins *i* for distribution *k* and interval *j*, is given by

6 $$\begin{aligned} \vec {p}_{kj}(\vec {\beta }_{j},\vec {\nu },\vec {R})&=\beta _{{\text {t}},j}\vec {T}_{{\text {t}},kj}^{t\text {-ch}}(\vec {\nu })+\beta _{\overline{{\mathrm{t}}},j}\vec {T}_{\overline{{\mathrm{t}}},kj}^{t\text {-ch}}(\vec {\nu })\nonumber \\&\quad +\beta _{\mathrm {t}\bar{\mathrm {t}}/{\text {tW}} ,j}\vec {T}_{kj}^{\mathrm {t}\bar{\mathrm {t}}/{\text {tW}} }(R_{j},\vec {\nu })\nonumber \\&\quad +\beta _{\text {W}{}{}/\text {Z}{}{}/{{\upgamma }{}{}} ^{*}\text {+jets},j}\vec {T}_{kj}^{\text {W}{}{}/\text {Z}{}{}/{{\upgamma }{}{}} ^{*}\text {+jets}}(R_{j},\vec {\nu })\nonumber \\&\quad +\beta _{\text {multijet},j}({\ell }{}{},r)\vec {T}_{kj}^{\text {multijet}}(R_{j}({\ell }{}{},r),\vec {\nu }), \end{aligned}$$

where $\vec {\nu }$ are the nuisance parameters, *R* the charge ratios of each background process, and $\beta$ the normalisations of the templates $\vec {T}$, which are independent per lepton flavour ${\ell }{}{}$ and category $r\in${2j1b, 3j2b} for the multijet templates. The profiling of systematic uncertainties leads to a correlation between the $t\text {-channel}$ top quark and antiquark yields in the same interval of about 20–30%. These correlations are propagated to the differential cross sections for each top quark charge, and are accounted for when calculating their sum and ratio.

Since the kinematic selection of electron events is restricted to $p_{\mathrm{T}} >35$ $\,\text {Ge}\text {V}$ and $|\eta |<1.48$, which is tighter than for muon events ($p_{\mathrm{T}} >26$ $\,\text {Ge}\text {V}$, $|\eta |<2.4$), the signal yields in the lowest interval of the lepton $p_{\mathrm{T}}$ and in the highest two intervals of the lepton rapidity spectra are estimated from the muon channel alone in the combined ${{\upmu }{}{}}/{\text {e}}$ fit.

## Validation of signal and background modelling

The distributions of the observables that are unfolded are validated by comparing the predictions to the data in a background-dominated as well as in a signal-enriched region before unfolding. Both regions are defined for events in the 2j1b category that also satisfy $m_{\mathrm{T}} (\text {W}{}{}) >50$ $\,\text {Ge}\text {V}$ to suppress the contribution from multijet production. The modelling of the $\mathrm {t}\bar{\mathrm {t}}{}{}/{\text{tW}}$ and $\text {W}{}{}/\text {Z}{}{}/{{\upgamma }{}{}} ^{*}\text {+jets}$ backgrounds is validated in a background-dominated region obtained from events having $\mathrm {BDT}_{t\text {-ch}} <0$. To validate the modelling of the $t\text {-channel}$ process, events are instead required to pass $\mathrm {BDT}_{t\text {-ch}} >0.7$, resulting in a sample enriched in signal events. These two regions and their selections are only defined and applied for validation purposes, and not used for measuring the differential cross sections for which the individual fit results are used in the unfolding instead.

**Fig. 5:**
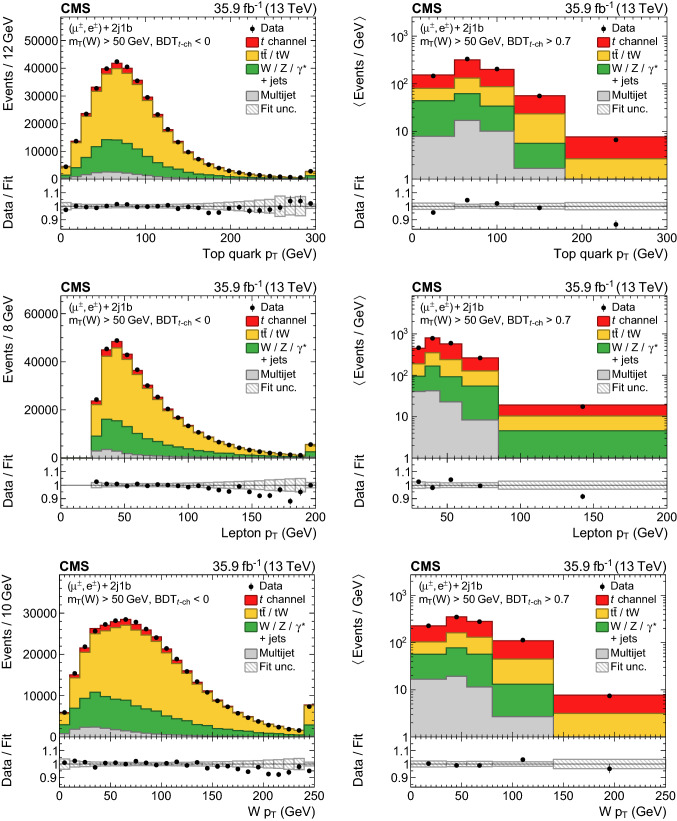
Distributions of the observables in a (left column) background-dominated and a (right column) signal-enriched region for events passing the 2 jets, 1 b  tag selection: (upper row) top quark $p_{\mathrm{T}}$; (middle row) charged lepton $p_{\mathrm{T}}$; (lower row) $\text {W}{}{}$  boson $p_{\mathrm{T}}$. Events in the muon and electron channels have been summed. The predictions have been scaled to the result of the inclusive ML fit and the hatched band displays the fit uncertainty. The plots on the left give the number of events per bin, while those on the right show the number of events per bin divided by the bin width. The lower panel in each plot gives the ratio of the data to the fit results. The right-most bins include the event overflows

**Fig. 6:**
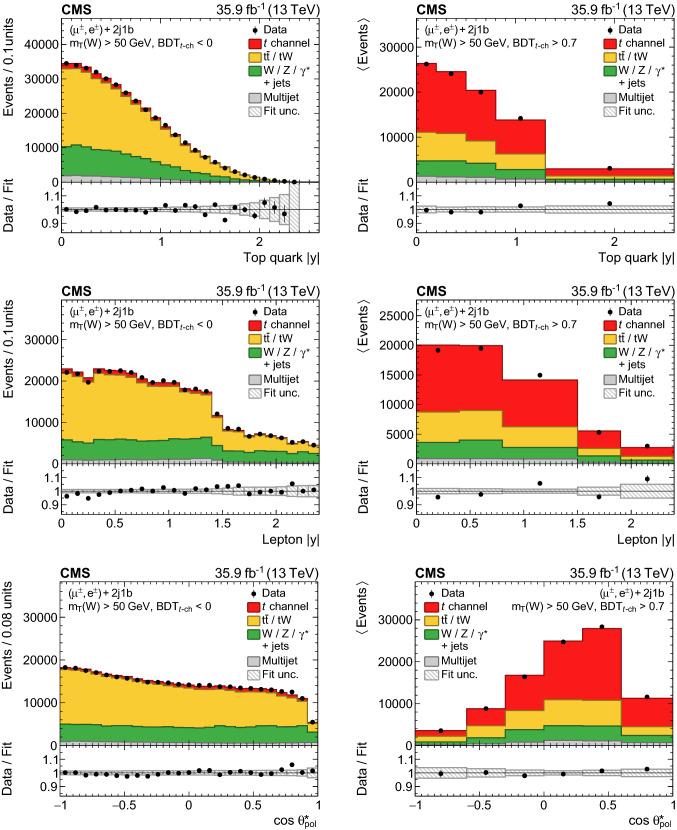
Distributions of the observables in a (left column) background-dominated and a (right column) signal-enriched region for events passing the 2 jets, 1 b  tag selection: (upper row) top quark rapidity; (middle row) charged lepton rapidity; (lower row) cosine of the top quark polarisation angle. Events in the muon and electron channels have been summed. The predictions have been scaled to the result of the inclusive ML fit and the hatched band displays the fit uncertainty. The plots on the left give the number of events per bin, while those on the right show the number of events per bin divided by the bin width. The lower panel in each plot gives the ratio of the data to the fit results

The resulting distributions in both regions for all six observables that are unfolded are shown in Figs. 5 and 6 after the predictions have been scaled to the inclusive fit result. Overall good agreement between the data and the fit result is observed in the background-dominated region, thus validating the modelling of the $\mathrm {t}\bar{\mathrm{t}}{}{}/{\text{tW}}$ and $\text {W}{}{}/\text {Z}{}{}/{{\upgamma }{}{}} ^{*}\text {+jets}$ backgrounds. In the signal region, reasonable agreement is also observed.

## Unfolding

The distributions from reconstructed events are affected by the detector resolution, selection efficiencies, and kinematic reconstruction, which lead to distortions with respect to the corresponding distributions at the parton or particle levels. The size of these effects varies with the event kinematics. In order to correct for these effects and determine the parton- and particle-level distributions, an unfolding method is applied to the reconstructed distributions. In this analysis, the tunfold algorithm [57] is chosen, which treats unfolding as a minimisation problem of the function

7 $$\begin{aligned} \chi ^2&=\left( \vec {y}-\varvec{R}\varvec{\epsilon }\vec {x}\right) ^\mathrm {T}\varvec{V}_{y}^{-1}\left( \vec {y}-\varvec{R}\varvec{\epsilon }\vec {x}\right) \nonumber \\&\quad +\underbrace{\tau ^2\left\Vert \varvec{L}(\vec {x}-\vec {x}_{0})\right\Vert ^2}_\text {regularisation}+\lambda \sum _{i}\left( \vec {y}-\varvec{R}\varvec{\epsilon }\vec {x}\right) _{i}, \end{aligned}$$

where $\vec {y}$ denotes the measured yields in data, $\varvec{V}_{y}$ is the covariance matrix of the measured yields, and $\vec {x}$ is the corresponding differential cross section at parton or particle level. The matrices $\varvec{R}$ and $\varvec{\epsilon }$ denote the transition probability and selection efficiencies, respectively, both estimated from simulation. The signal yields and covariances are estimated through ML fits using the $m_{\mathrm{T}} (\text {W}{}{})$, $\mathrm {BDT}_{\mathrm {t}\bar{\mathrm {t}}/\text {W}{}{}}$, and $\mathrm {BDT}_{t\text {-ch}}$ distributions, as detailed in Sect. 6.

A penalty term, based on the curvature of the unfolded spectrum [58, 59] encoded in the matrix $\varvec{L}$, is added in the minimisation to suppress oscillating solutions originating from amplified statistical fluctuations. This “regularisation” procedure has a strength $\tau$ that is chosen to minimise the global correlation between the unfolded bins. The “bias vector” $\vec {x}_{0}$ is set to the expected spectrum from simulation. Pseudo-experiments using simulated data are performed to verify that the unfolding method estimates the uncertainties correctly, while keeping the regularisation bias at a minimum. No regularisation is applied when unfolding the lepton $p_{\mathrm{T}}$ and rapidity spectra since the migrations between bins are found to be negligible. The overall normalisation of the unfolded spectrum is determined by performing a simultaneous minimisation with respect to the Lagrange multiplier $\lambda$.

The parton-level top quark in simulation is defined as the generated on-shell top quark after quantum electrodynamic (QED) and QCD radiation, taking into account the intrinsic transverse momentum of initial-state partons. Events are required to contain either a muon or an electron from the top quark decay chain. This also includes muons or electrons from intermediately produced ${{\uptau }{}{}}$ leptons. In such events, the $\text {W}{}{}$  boson is chosen to be the direct daughter of the top quark. The spectator quark is selected from among the light quarks after QED and QCD radiation that are not products of the top quark decay. In case of ambiguities arising from initial-state radiation, the spectator quark that minimises the $p_{\mathrm{T}}$ of the combined spectator quark and top quark system is chosen.

The top quark at the particle level (called “pseudo top quark”) is defined in simulated events by performing an event reconstruction based on the set of stable simulated particles after hadronisation [60]. In the context of this study, all particles with a lifetime of more than 30$\,\text {ps}$ are considered stable. So-called “dressed” muons and electrons are constructed by accounting for the additional momenta carried by photons within a cone of $\varDelta R<0.1$ around the corresponding prompt lepton that do not originate from hadronisation products. The ${\vec p}_{\mathrm{T}}^{\text {miss}}$ is defined as the summed momentum of all prompt neutrinos in the event. Jets at the particle level are clustered from all stable particles excluding prompt muons, prompt electrons, prompt photons, and all neutrinos using the anti-$k_{\mathrm{T}}$ algorithm with a distance parameter of $R=0.4$. From these objects, a pseudo top quark is reconstructed by first solving for the unknown neutrino $p_{z}$ momentum, which is identical to the top quark reconstruction procedure applied to data, as described in Sect. 4. Events containing a single dressed muon or electron with $p_{\mathrm{T}} >26$ $\,\text {Ge}\text {V}$ and $|\eta |<2.4$, together with two jets with $p_{\mathrm{T}} >40$ $\,\text {Ge}\text {V}$ and $|\eta |<4.7$, are considered at the particle level. Jets that are closer than $\varDelta R=0.4$ to the selected dressed muon or electron are ignored. The jet that yields a top quark mass closest to 172.5$\,\text {Ge}\text {V}$ is assumed to come from the top quark decay, while the other jet is taken as the spectator jet.

The size of the binning intervals are chosen to minimise the migrations between the reconstructed bins while retaining sensitivity to the shapes of the distributions. The stability (purity) is defined as the probability that the parton- or particle-level (reconstructed) values of an observable within a certain range also have their reconstructed (parton-/particle-level) counterparts in the same range. Both quantities are found to be greater than or equal to 50% in most bins of all distributions, with the exception of a few bins at the parton level where purity and stability drop to 40%, and the first two bins of the polarisation angle distribution at the parton level where both quantities drop to about 25%. The stability and purity values are about 10% larger for the particle-level distributions than for the parton-level ones. The acceptance times efficiency for selecting $t\text {-channel}$ single top quark events at the detector level is found to be 2–8 (20–30)% for muon events and 1–5 (10–20)% for electron events with respect to the parton (particle) level across the unfolding bins.

## Systematic uncertainties

The measurements are affected by various sources of systematic uncertainty. For each systematic variation, new templates and response matrices are derived. Systematic variations can create correlations between the $t\text {-channel}$ top quark and antiquark yields since both yields are estimated simultaneously from data through an ML fit, as described in Sect. 6.

The following experimental systematic uncertainties are profiled in the ML fit.

- Background composition: As described in Sect. 6, the $\text {Z}{}{}/{{\upgamma }{}{}} ^{*}\text {+jets}$ and $\text {W}{}{} \text {+jets}$ processes and the $\mathrm {t}\bar{\mathrm {t}}$ and ${\text{tW}}$ processes are separately grouped together in the ML fit. The ratios of the $\text {Z}{}{}/{{\upgamma }{}{}} ^{*}\text {+jets}$ to the $\text {W}{}{} \text {+jets}$ yields and the $\mathrm {t}\bar{\mathrm {t}}$ to the ${\text{tW}}$ yields are assigned a ${\pm }20$% uncertainty. This covers the uncertainty in the small $\text {Z}{}{}/{{\upgamma }{}{}} ^{*}\text {+jets}$ and ${\text{tW}}$ yields, for which the analysis has little sensitivity.
- Multijet shape estimation: The multijet event distributions are estimated from data by inversion of the muon isolation criterion or the electron identification criteria. The uncertainty in the shape of these distributions is estimated by varying the criteria. The requirement on the muon isolation parameter in the sideband region is modified from $I_{\mathrm{rel}}^{{{\upmu }{}{}}} >20$% to either $20< I_{\mathrm{rel}}^{{{\upmu }{}{}}} < 40$% or $I_{\mathrm{rel}}^{{{\upmu }{}{}}} > 40$%, and the electron isolation parameter to either $I_\text {rel}^{{\text {e}}} < 30$% or $I_\text {rel}^{{\text {e}}} > 5.88$%, while inverting the identification criteria. Another variation is done by requiring electrons in the sideband region to explicitly pass or fail the photon conversion criterion, which is also part of the electron identification requirement.
- Efficiency of b  tagging and misidentification: The scale factors used to reweight the b  tagging and misidentification efficiencies in simulation to the ones estimated from data are varied within their uncertainties based on the true flavour of the selected jets [49].
- Jet energy scale and resolution: The jet energy scale and resolution corrections are varied within their uncertainties [61]. The shifts induced in the jet momenta are propagated to ${\vec p}_{\mathrm{T}}^{\text {miss}}$ as well.
- Unclustered energy: The contributions to $p_{\mathrm{T}} ^\text {miss}$ of PF candidates that have not been clustered into jets are varied within their respective energy resolutions [62].
- Pileup: The simulated distribution of pileup interactions is modified by shifting the total inelastic ${\text {p}{}{}} {\text {p}{}{}}$ cross section by ${\pm }5\%$ [63].
- Lepton efficiencies: The scale factors that account for differences in the lepton selection and reconstruction efficiencies between data and simulation are varied within their uncertainties [23, 46].

The systematic uncertainties in the theoretical modelling of the simulated samples are estimated by using new templates and response matrices in the ML fit and unfolding for each variation. For each uncertainty source, the maximum difference of the up/down variations with the result using the nominal templates and response matrix is taken as the estimated uncertainty per bin. These are added in quadrature to the experimental uncertainty per bin.

The following sources of theoretical uncertainty have been evaluated.

- Modelling of top quark $p_{\mathrm{T}}$ in $\mathrm {t}\bar{\mathrm {t}}$ events: Differential cross section measurements of $\mathrm {t}\bar{\mathrm {t}}$ production by CMS [64, 65] have shown that the $p_{\mathrm{T}}$ spectrum of top quarks in $\mathrm {t}\bar{\mathrm {t}}$ events is significantly softer than predicted by NLO simulations. To correct for this effect, simulated $\mathrm {t}\bar{\mathrm {t}}$ events are reweighted according to the scale factors derived from measurements at 13$\,\text {Te}\text {V}$  [65]. The difference in the predictions when using the default $\mathrm {t}\bar{\mathrm {t}}$ simulation sample is taken as an additional uncertainty.
- Top quark mass: The nominal top quark mass of 172.5$\,\text {Ge}\text {V}$ is modified by ${\pm }0.5$ $\,\text {Ge}\text {V}$ in the simulation [66]. The difference with respect to the nominal simulation results is taken as the corresponding uncertainty.
- Parton distribution functions: The effect of the uncertainty in the PDFs is estimated by reweighting the simulated events using the recommended variations in the NNPDF3.0 NLO set, including a variation of $\alpha _S$ [35]. The reweighting is performed using precomputed weights stored in the event record by the matrix element generator [67].
- Renormalisation/factorisation scales: A reweighting procedure similar to that used for the PDFs is carried out on simulated $t\text {-channel}$, $\text {W}{}{} \text {+jets}$, and $\mathrm {t}\bar{\mathrm {t}}$ simulated events to estimate the effect of the uncertainties in the renormalisation and factorisation scales. The weights correspond to independent variations by factors of 0.5 and 2 in the scales with respect to their nominal values. The envelope of all possible combinations of up-varied/down-varied scales with the exception of the extreme up/down combinations is considered as an uncertainty. This uncertainty is evaluated independently for the $t\text {-channel}$, $\text {W}{}{} \text {+jets}$, and $\mathrm {t}\bar{\mathrm {t}}$ simulated event samples.
- Parton shower: The uncertainties in the parton shower simulation are evaluated by comparing the nominal samples to dedicated samples with varied shower parameters. For $t\text {-channel}$ single top quark production, the differences with respect to samples with a varied factorisation scale by a factor of 0.5 or 2 or with a varied powheg $h_\text {damp}$ parameter are taken as two independent uncertainties. For the simulated $\mathrm {t}\bar{\mathrm {t}}$ samples, the variation of the factorisation scale in both initial- and final-state radiation, and the $h_\text {damp}$ parameter are evaluated as three independent uncertainties.
- Underlying event tune: The impact of uncertainties arising from the CUETP8M2T4 underlying event tune [30] used in the simulation of $\mathrm {t}\bar{\mathrm {t}}$ events is evaluated using dedicated samples with the tune varied within its uncertainties.
- Colour reconnection: The default model of colour reconnection in pythia is based on multiple-particle interactions (MPI) with early resonance decays switched off. An uncertainty in the choice of this model is taken into account by repeating the measurement using three alternative models of colour reconnection in the simulation of $t\text {-channel}$ single top quark and $\mathrm {t}\bar{\mathrm {t}}$ production: the MPI-based scheme with early resonance decays switched on, a gluon-move scheme [68], and a QCD-inspired scheme [69].
- Fragmentation model: The fragmentation of b quarks, modelled by the Bowler-Lund function [70], is varied within its uncertainties for $t\text {-channel}$ single top quark and $\mathrm {t}\bar{\mathrm {t}}$ production. Additionally, the impact when using the Peterson model [71] for b quark fragmentation instead is assessed.

In addition, an uncertainty of ${\pm }2.5\%$ in the measurement of the integrated luminosity of the data set [22] is taken into account by scaling the evaluated covariance matrix per observable accordingly.

## Results

**Fig. 7:**
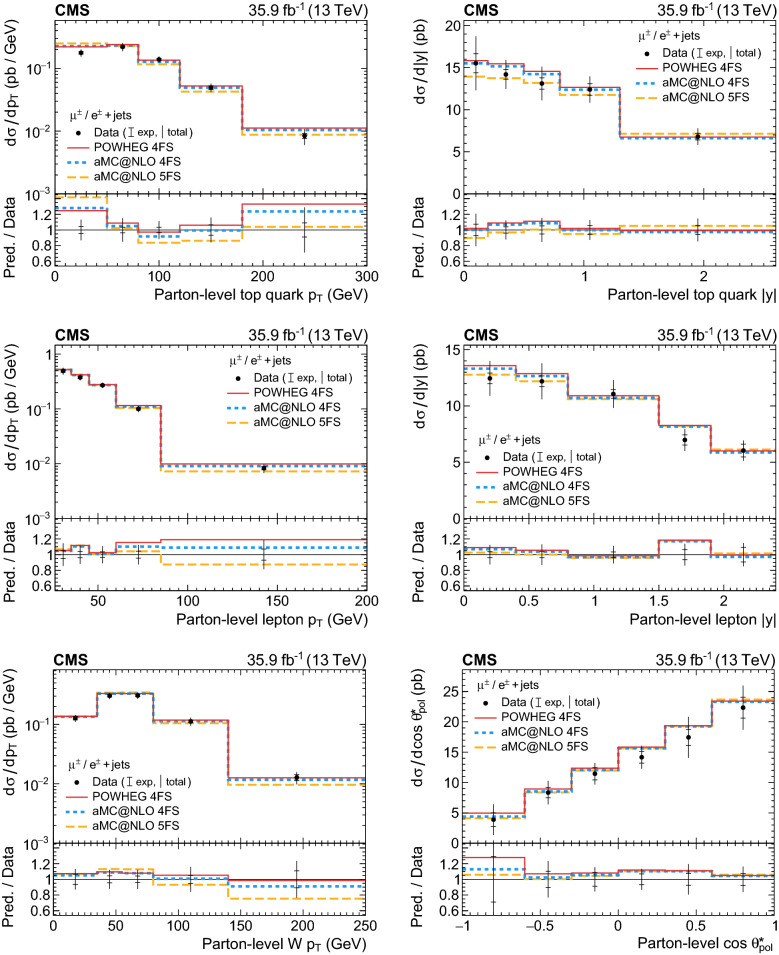
Differential cross sections for the sum of $t\text {-channel}$ single top quark and antiquark production at the parton level: (upper row) top quark $p_{\mathrm{T}}$ and rapidity; (middle row) charged lepton $p_{\mathrm{T}}$ and rapidity; (lower left) $\text {W}{}{}$  boson $p_{\mathrm{T}}$; (lower right) cosine of the top quark polarisation angle. The total uncertainty is indicated by the vertical lines, while horizontal bars indicate the statistical and experimental uncertainties, which have been profiled in the ML fit, and thus exclude the uncertainties in the theoretical modelling and the luminosity. Three different predictions from event generators are shown by the solid, dashed, and dotted lines. The lower panels show the ratios of the predictions to the data

**Fig. 8:**
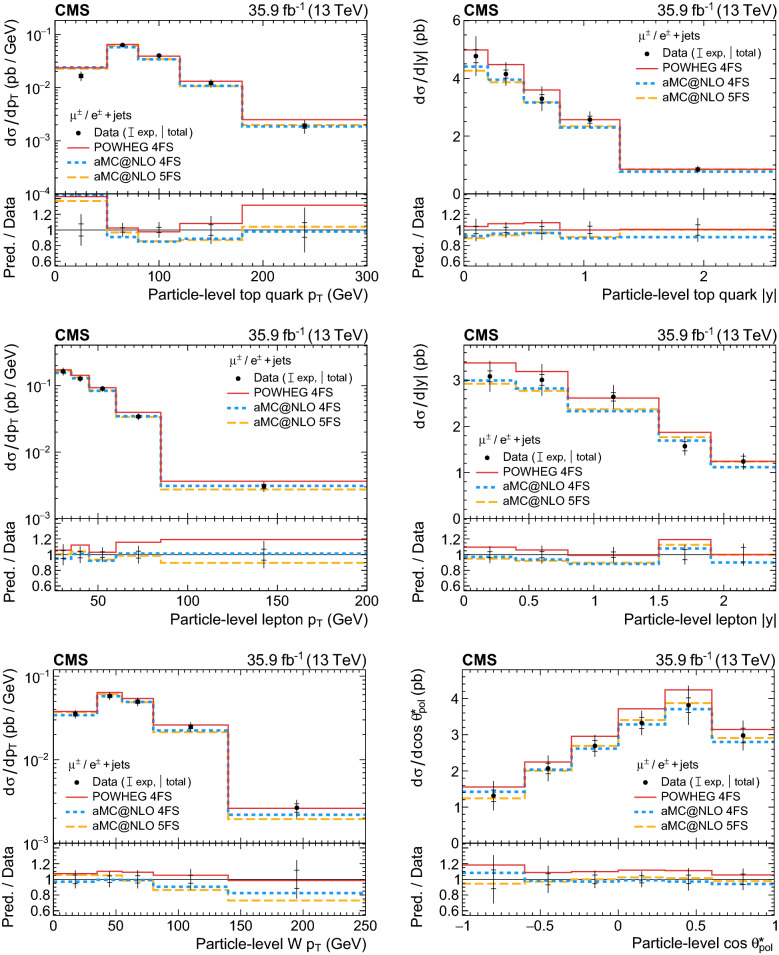
Differential cross sections for the sum of $t\text {-channel}$ single top quark and antiquark production at the particle level: (upper row) top quark $p_{\mathrm{T}}$ and rapidity; (middle row) charged lepton $p_{\mathrm{T}}$ and rapidity; (lower left) $\text {W}{}{}$  boson $p_{\mathrm{T}}$; (lower right) cosine of the top quark polarisation angle. The total uncertainty is indicated by the vertical lines, while horizontal bars indicate the statistical and experimental uncertainties, which have been profiled in the ML fit, and thus exclude the uncertainties in the theoretical modelling and the luminosity. Three different predictions from event generators are shown by the solid, dashed, and dotted lines. The lower panels show the ratios of the predictions to the data

**Fig. 9:**
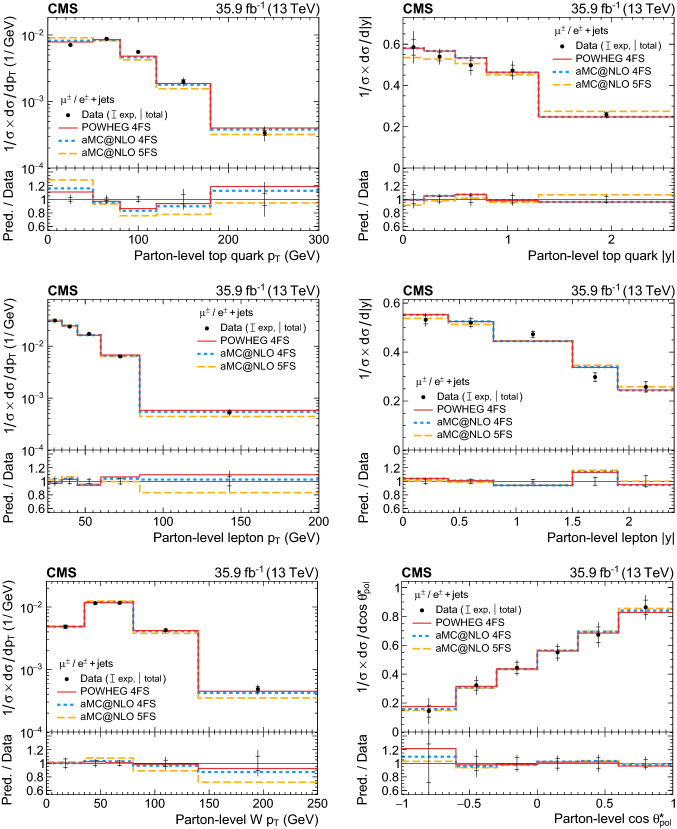
Normalised differential cross sections for the sum of $t\text {-channel}$ single top quark and antiquark production at the parton level: (upper row) top quark $p_{\mathrm{T}}$ and rapidity; (middle row) charged lepton $p_{\mathrm{T}}$ and rapidity; (lower left) $\text {W}{}{}$  boson $p_{\mathrm{T}}$; (lower right) cosine of the top quark polarisation angle. The total uncertainty is indicated by the vertical lines, while horizontal bars indicate the statistical and experimental uncertainties, which have been profiled in the ML fit, and thus exclude the uncertainties in the theoretical modelling. Three different predictions from event generators are shown by the solid, dashed, and dotted lines. The lower panels show the ratios of the predictions to the data

**Fig. 10:**
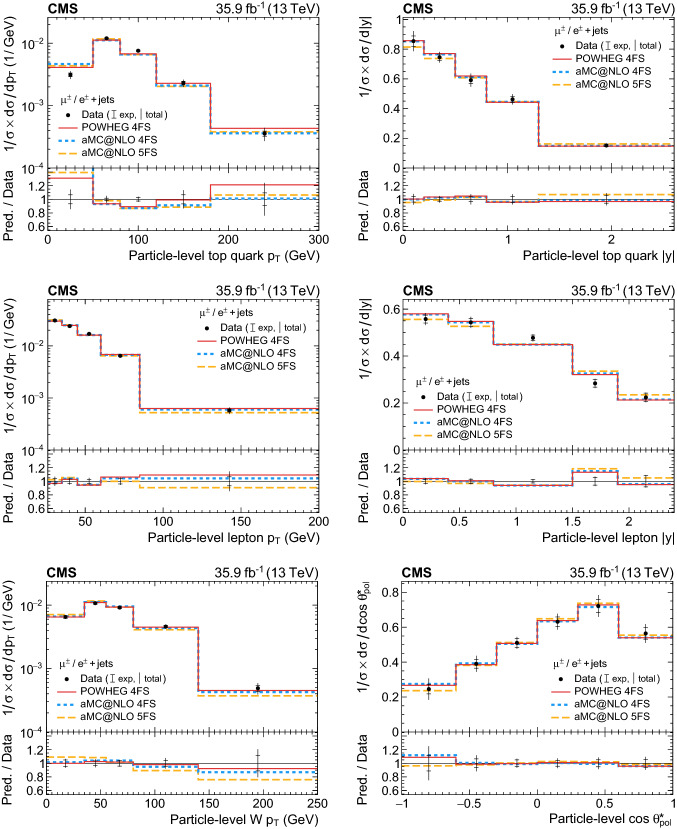
Normalised differential cross sections for the sum of $t\text {-channel}$ single top quark and antiquark production at the particle level: (upper row) top quark $p_{\mathrm{T}}$ and rapidity; (middle row) charged lepton $p_{\mathrm{T}}$ and rapidity; (lower left) $\text {W}{}{}$  boson $p_{\mathrm{T}}$; (lower right) cosine of the top quark polarisation angle. The total uncertainty is indicated by the vertical lines, while horizontal bars indicate the statistical and experimental uncertainties, which have been profiled in the ML fit, and thus exclude the uncertainties in the theoretical modelling. Three different predictions from event generators are shown by the solid, dashed, and dotted lines. The lower panels show the ratios of the predictions to the data

Differential cross sections of $t\text {-channel}$ single top quark production as a function of the top quark $p_{\mathrm{T}}$, rapidity, and polarisation angle, the $p_{\mathrm{T}}$ and rapidity of the charged lepton (muon or electron) that originates from the top quark decay, and the $p_{\mathrm{T}}$ of the $\text {W}{}{}$  boson from the top quark decay are presented in Figs. 7 and 8 at the parton and particle levels, respectively. The normalised differential cross sections of the same observables at the parton and particle levels are provided in Figs. 9 and 10. The total uncertainty is indicated by the vertical lines, while horizontal bars indicate the statistical and experimental uncertainties, which have been profiled in the ML fit, and thus exclude the uncertainties in the theoretical modelling and the luminosity. The differential cross sections refer to $t\text {-channel}$ single top quark production where the top quark decays semileptonically (into either muon or electron) including events where the charged lepton stems from an intermediate ${{\uptau }{}{}}$ lepton decay. The results are compared to the predictions by the powheg generator interfaced with pythia in the 4FS and the MadGraph 5_amc@nlo generator interfaced with pythia in the 4FS and 5FS.

An overall good agreement of the results with the predictions from the 4FS is observed, except for a slight deviation at low top quark $p_{\mathrm{T}}$. The predictions from the 5FS for the top quark and $\text {W}{}{}$  boson $p_{\mathrm{T}}$ distributions do not agree as well with the data.

**Fig. 11:**
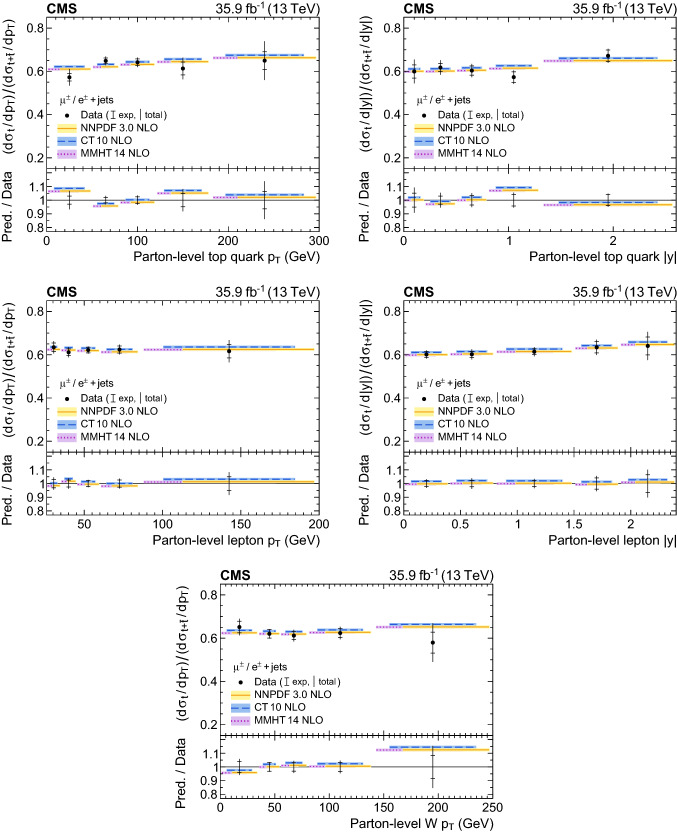
Ratio of the top quark to the sum of the top quark and antiquark $t\text {-channel}$ differential cross section at the parton level: (upper row) top quark $p_{\mathrm{T}}$ and rapidity; (middle row) charged lepton $p_{\mathrm{T}}$ and rapidity; (lower row) $\text {W}{}{}$  boson $p_{\mathrm{T}}$. The total uncertainty is indicated by the vertical lines, while horizontal bars indicate the statistical and experimental uncertainties, which have been profiled in the ML fit, and thus exclude the uncertainties in the theoretical modelling. Predictions from three different PDF sets are shown by the solid, dashed, and dotted lines. The lower panels show the ratios of the predictions to the data

**Fig. 12:**
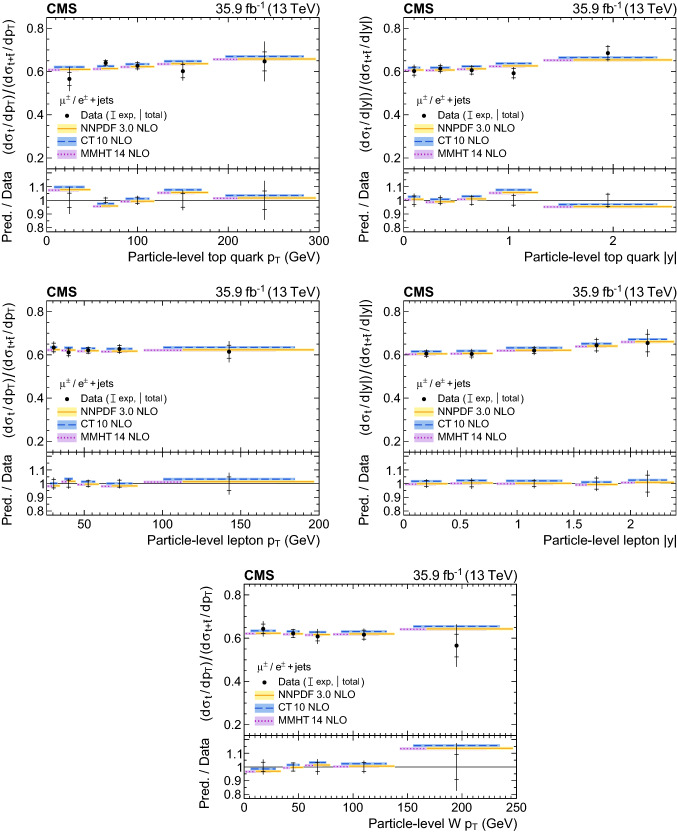
Ratio of the top quark to the sum of the top quark and antiquark $t\text {-channel}$ differential cross section at the particle level: (upper row) top quark $p_{\mathrm{T}}$ and rapidity; (middle row) charged lepton $p_{\mathrm{T}}$ and rapidity; (lower row) $\text {W}{}{}$  boson $p_{\mathrm{T}}$. The total uncertainty is indicated by the vertical lines, while horizontal bars indicate the statistical and experimental uncertainties, which have been profiled in the ML fit, and thus exclude the uncertainties in the theoretical modelling. Predictions from three different PDF sets are shown by the solid, dashed, and dotted lines. The lower panels show the ratios of the predictions to the data

Differential ratios of the top quark production rates to the sum of the top quark and antiquark rates as a function of the top quark $p_{\mathrm{T}}$ and rapidity, the $p_{\mathrm{T}}$ and rapidity of the charged lepton, and the $\text {W}{}{}$  boson $p_{\mathrm{T}}$ are presented in Figs. 11 and 12 at the parton and particle levels, respectively. It is found that the standard definition of the charge ratio in the literature, i.e. $\sigma _{{\text {t}}}/\sigma _{{ {{\text {t}}}}}$, can yield large variances when the precision in certain intervals of the differential cross section for the top antiquark is low. Therefore, the charge ratio is defined as $\sigma _{{\text {t}}}/\sigma _{{\text {t+t}}}$ in this paper. The ratios have been calculated from the measured cross sections at the parton and particle levels, while accounting for correlations between the top quark and antiquark spectra, as detailed in Sects. 6 and 9. The resulting charge ratios are compared to the predictions by the NNPDF3.0 NLO, MMHT14 NLO [72], and CT10 NLO PDF sets, which have been calculated using the powheg signal sample—generated in the 4FS and interfaced with pythia. The uncertainty bands shown in Figs. 11 and 12 represent the total uncertainty from varying the corresponding PDF eigenvectors and $\alpha _S$. Within the uncertainties, the measured charge ratios are in good agreement with the predictions from all three PDF sets.

The spin asymmetry, sensitive to the top quark polarisation, is determined from the differential cross section as a function of the polarisation angle at the parton level (Fig. 7, lower right). A linear $\chi ^{2}$-based fit, assuming the expected functional dependence given in Eq. (2), is used to take the correlations between the unfolded bins into account. The measured spin asymmetry in the muon and electron channel and their combination is given in Table 2.

**Table 2:** The measured spin asymmetry in the muon and electron channel and their combination. A breakdown of the systematic uncertainties is also provided. Minor systematic uncertainties (lepton efficiencies, pileup, and unclustered energy) have been grouped into the “Others” category

	Central values	$A_{{{\upmu }{}{}}}$	$A_{{\text {e}}}$	$A_{{{\upmu }{}{}} \text {+}{\text {e}}}$
		0.403	0.446	0.440
Profiled uncertainties	Statistical	${\pm }$0.029	${\pm }$0.038	${\pm }$0.024
	$\mathrm{t\overline{t}/tW}$ normalisation	${\pm }$0.010	${\pm }$0.007	${\pm }$0.007
	$\text {W}{}{}/\text {Z}{}{}/{{\upgamma }{}{}} ^{*}\text {+jets}$ normalisation	${\pm }$0.012	${\pm }$0.011	${\pm }$0.012
	Multijet normalisation	${<}$0.001	${<}$0.001	${\pm }$0.003
	Multijet shape	${<}$0.001	${\pm }$0.006	${<}$0.001
	Jet energy scale/resolution	${\pm }$0.008	${<}$0.001	${<}$0.001
	b tagging efficiencies/misidentification	${<}$0.001	${\pm }$0.009	${\pm }$0.004
	Others	${<}$0.001	${\pm }$0.003	${\pm }$0.005
Theoretical uncertainties	Top quark mass	${\pm }$0.033	${\pm }$0.063	${\pm }$0.044
	PDF+$\alpha _S$	${\pm }$0.011	${\pm }$0.009	${\pm }$0.011
	*t* channel renorm./fact. scales	${\pm }$0.013	${\pm }$0.018	${\pm }$0.020
	*t* channel parton shower	${\pm }$0.030	${\pm }$0.008	${\pm }$0.014
	$\mathrm {t}\bar{\mathrm {t}}$ renorm./fact. scales	${\pm }$0.008	${\pm }$0.019	${\pm }$0.017
	$\mathrm {t}\bar{\mathrm {t}}$ parton shower	${\pm }$0.031	${\pm }$0.037	${\pm }$0.033
	$\mathrm {t}\bar{\mathrm {t}}$ underlying event tune	${<}$0.001	${\pm }$0.014	${\pm }$0.014
	$\mathrm {t}\bar{\mathrm {t}}$ $p_{\mathrm{T}}$ reweighting	${<}$0.001	${\pm }$0.010	${\pm }$0.009
	$\text {W}{}{} \text {+jets}$ renorm./fact. scales	${<}$0.001	${\pm }$0.019	${\pm }$0.014
	Color reconnection	${\pm }$0.036	${\pm }$0.056	${\pm }$0.031
	Fragmentation model	${\pm }$0.011	${\pm }$0.011	${\pm }$0.011
Profiled uncertainties only (statistical+experimental)		${\pm }$0.041	${\pm }$0.047	${\pm }$0.031
Total uncertainties		${\pm }$0.071	${\pm }$0.099	${\pm }$0.070

The measured asymmetries are in good agreement with the predicted SM value of 0.436, found using powheg at NLO, with a negligible uncertainty. Good agreement is also found with a corresponding measurement by the ATLAS Collaboration at $\sqrt{s}=8$ $\,\text {Te}\text {V}$  [17]. This measurement is found to be more precise than a previous analysis of the spin asymmetry at $\sqrt{s}=8$ $\,\text {Te}\text {V}$ by the CMS Collaboration [9]. In particular, the deviation found therein, corresponding to 2.0 standard deviations, is not seen.

## Summary

Differential cross sections for $t\text {-channel}$ single top quark and antiquark production in proton–proton collisions at $\sqrt{s}=13$ $\,\text {Te}\text {V}$ have been measured by the CMS experiment at the LHC using a sample of proton–proton collision data, corresponding to an integrated luminosity of 35.9$\,\text {fb}^{-1}$. The cross sections are determined as a function of the top quark transverse momentum ($p_{\mathrm{T}}$), rapidity, and polarisation angle, the charged lepton $p_{\mathrm{T}}$ and rapidity, and the $p_{\mathrm{T}}$ of the $\text {W}{}{}$  boson from the top quark decay. In addition, the charge ratio has been measured as a function of the top quark, charged lepton, and $\text {W}{}{}$  boson kinematic observables. Events containing one muon or electron and two or three jets are used. The single top quark and antiquark yields are determined through maximum-likelihood fits to the data distributions. The differential cross sections are then obtained at the parton and particle levels by unfolding the measured signal yields.

The results are compared to various next-to-leading-order predictions, and found to be in good agreement. Furthermore, the top quark spin asymmetry, which is sensitive to the top quark polarisation, has been measured using the differential cross section as a function of the top quark polarisation angle at the parton level. The resulting value of $0.440 \pm 0.070$ is in good agreement with the standard model prediction.

These results demonstrate a good understanding of the underlying electroweak production mechanism of single top quarks at $\sqrt{s}=13$ $\,\text {Te}\text {V}$ and in particular of the electroweak vector−axial-vector coupling predicting highly polarized top quarks. Lastly, the differential charge ratios, sensitive to the ratio of the up to down quark content of the proton, are found to be consistent with the predictions by various sets of parton distribution functions.

## Data Availability

This manuscript has no associated data or the data will not be deposited. [Authors’ comment: Release and preservation of data used by the CMS Collaboration as the basis for publications is guided by the CMS policy as written in its document “CMS data preservation, re-use and open access policy” (https://cms-docdb.cern.ch/cgi-bin/PublicDocDB/RetrieveFile?docid=6032&filename=CMSDataPolicyV1.2.pdf&version=2).]
